# CD99–PTPN12 Axis Suppresses Actin Cytoskeleton-Mediated Dimerization of Epidermal Growth Factor Receptor

**DOI:** 10.3390/cancers12102895

**Published:** 2020-10-09

**Authors:** Kyoung-Jin Lee, Yuri Kim, Min Seo Kim, Hyun-Mi Ju, Boyoung Choi, Hansoo Lee, Dooil Jeoung, Ki-Won Moon, Dongmin Kang, Jiwon Choi, Jong In Yook, Jang-Hee Hahn

**Affiliations:** 1 Department of Anatomy and Cell Biology, School of Medicine, Kangwon National University, Chuncheon 24341, Korea; jin.lee@supadelixir.com (K.-J.L.); yuri8686@sunmoon.ac.kr (Y.K.); ms.kim@supadelixir.com (M.S.K.); hm.ju@supadelixir.com (H.-M.J.); boyoung630@naver.com (B.C.); 2 Department of Biological Sciences, College of Natural Sciences, Kangwon National University, Chuncheon 24341, Korea; hslee@kangwon.ac.kr; 3 Department of Biochemistry, College of Natural Sciences, Kangwon National University, Chuncheon 24341, Korea; jeoungd@kangwon.ac.kr; 4 Department of Rheumatology, Kangwon National University Hospital, Chuncheon 24289, Korea; kiwonmoon@kangwon.ac.kr; 5 Department of Life Science, Ewha Womans University, Seoul 03760, Korea; dkang@ewha.ac.kr; 6 Met Life Sciences Co., Ltd., Seoul 03722, Korea; edccjw@gmail.com (J.C.); jiyook@yuhs.ac (J.I.Y.); 7 Department of Oral Pathology, Oral Cancer Research Institute, College of Dentistry, Yonsei University, Seoul 03722, Korea

**Keywords:** actin cytoskeletal reorganization, breast cancer, CD99 agonist, EGFR dimerization, endocytosis, FAK dephosphorylation, PTPN12, Rac1, RhoA, tripeptide

## Abstract

**Simple Summary:**

The epidermal growth factor receptor (EGFR) is activated through growth factor-dependent dimerization accompanied by functional reorganization of the actin cytoskeleton. Lee et al. demonstrate that CD99 activation by agonist ligands inhibits epidermal growth factor (EGF)-induced EGFR dimerization through impairment of cytoskeletal reorganization by protein tyrosine phosphatase non-receptor type 12 (PTPN12)-dependent c-Src/focal adhesion kinase (FAK) inactivation, thereby suppressing breast cancer growth.

**Abstract:**

The epidermal growth factor receptor (EGFR), a member of ErbB receptor tyrosine kinase (RTK) family, is activated through growth factor-induced reorganization of the actin cytoskeleton and subsequent dimerization. We herein explored the molecular mechanism underlying the suppression of ligand-induced EGFR dimerization by CD99 agonists and its relevance to tumor growth in vivo. Epidermal growth factor (EGF) activated the formation of c-Src/focal adhesion kinase (FAK)-mediated intracellular complex and subsequently induced RhoA-and Rac1-mediated actin remodeling, resulting in EGFR dimerization and endocytosis. In contrast, CD99 agonist facilitated FAK dephosphorylation through the HRAS/ERK/PTPN12 signaling pathway, leading to inhibition of actin cytoskeletal reorganization via inactivation of the RhoA and Rac1 signaling pathways. Moreover, CD99 agonist significantly suppressed tumor growth in a BALB/c mouse model injected with MDA-MB-231 human breast cancer cells. Taken together, these results indicate that CD99-derived agonist ligand inhibits epidermal growth factor (EGF)-induced EGFR dimerization through impairment of cytoskeletal reorganization by PTPN12-dependent c-Src/FAK inactivation, thereby suppressing breast cancer growth.

## 1. Introduction

Many studies have focused on uncovering the molecular basis of epidermal growth factor receptor (EGFR) activation and its implication in tumor development and progression. EGFR is activated by ligand binding, which induces sequentially their conformational change, auto- and trans-phosphorylation, dimerization, and internalization [1,2,3]. Structural study demonstrated that EGFR can be multimerized through a specific region of subdomain IV of the extracellular domain [4]. On the other hand, an increasing number of studies have suggested different aspects of EGFR activation. An inactive pre-formed dimer of EGFR without ligand was identified at the cell surface, which undergoes conformational changes during the activation process by stimulated ligand binding [5,6,7]. In spite of various aspects of the regulation of EGFR activation, dimerization of EGFR is a common feature required for its activation and transmission of downstream signals in tumorigenesis.

Non-receptor type 12 protein tyrosine phosphatase (PTPN12) acts as a core regulator in actin cytoskeleton-mediated modulation of growth factor receptor dynamics [8]. PTPN12 controls Rho GTPase activity by suppressing the interaction of p120 catenin with guanine nucleotide exchange factor VAV2 [9,10,11,12]. More recently, it was demonstrated that ephrin receptor (Eph) signaling depends on cytoskeletal reorganization to form polymeric assembly of the receptors and that PTPN12 contrarily downregulates EphA3 activity by inhibiting actin cytoskeletal remodeling. Interestingly, PTPN12 acts as a tumor suppressor which regulates the activities of multiple oncogenic tyrosine kinases, including EGFR, human epidermal growth factor receptor 2 (HER2), and platelet-derived growth factor receptor-β (PDGFRβ), and its deficiency is identified in several carcinomas [13,14]. Loss of PTPN12 promotes in vivo tumor progression of the implanted breast cancer cells, which express a constitutively active form of ErbB2 (CA-ErbB2), and correlates with the impaired feedback regulation of RTKs, thereby resulting in their aberrant activation [15,16]. In this regard, the expression levels of PTPN12 correlate inversely with poor prognosis of hepatocellular carcinoma [17]. Therefore, these results suggest that PTPN12 may play a role in regulation of growth factor receptor dimerization through actin cytoskeleton remodeling.

CD99 is a 32-kDa heavily O-glycosylated type I transmembrane protein [18]. CD99 is expressed on the surface of nearly all normal cell types including thymocytes, peripheral T cells, hematopoietic cells, and also several tumors including Ewing’s sarcoma [19,20]. It has been known that CD99 is implicated in various cellular processes including differentiation, apoptosis, homotypic aggregation, and proliferation of lymphocytes, extravasation of leukocytes, transport of several transmembrane proteins, and apoptosis of tumor cells [21,22,23]. We previously reported that CD99CRIII3, a CD99 agonistic peptide ligand, activated PKA-SHP2-HRAS-ERK1/2 signal transduction pathway, which led to upregulation of PTPN12 expression and interaction with its downstream targets, FAK and PIN1, resulting in dephosphorylation of FAK at Y397 [24]. Consistent with our study, CD99 regulates contact strength and motility of osteosarcoma cells through inhibition of the expression of coiled-coil containing protein kinase 2 (ROCK2), which is a crucial mediator of actin cytoskeleton remodeling [25]. Inhibition of ROCK2 expression leads to a significant decrease in expression and phosphorylation of Ezrin, thereby collapsing the crosslinks between the plasma membrane and cytoskeleton. These results prompted us to hypothesize that CD99 activation can regulate actin cytoskeleton dynamics through the PTPN12/FAK/Rho/Rac axis, thereby suppressing EGFR dimerization and activation.

In this study, we examined the molecular mechanism through which CD99 agonist ligand suppresses ligand-induced dimerization and internalization of EGFR in breast carcinoma cells. Our study suggests that CD99-derived agonist ligands inhibit EGF-induced EGFR dimerization through impairing RhoA-Rac1 signaling-mediated reorganization of the actin cytoskeleton, thereby contributing to the suppression of breast cancer growth.

## 2. Results

### 2.1. Epidermal Growth Factor Stimulates Dimerization and Activation of the Epidermal Growth Factor Receptor through c-Src/FAK-Mediated Actin Cytoskeleton Remodeling

**Figure 1:**
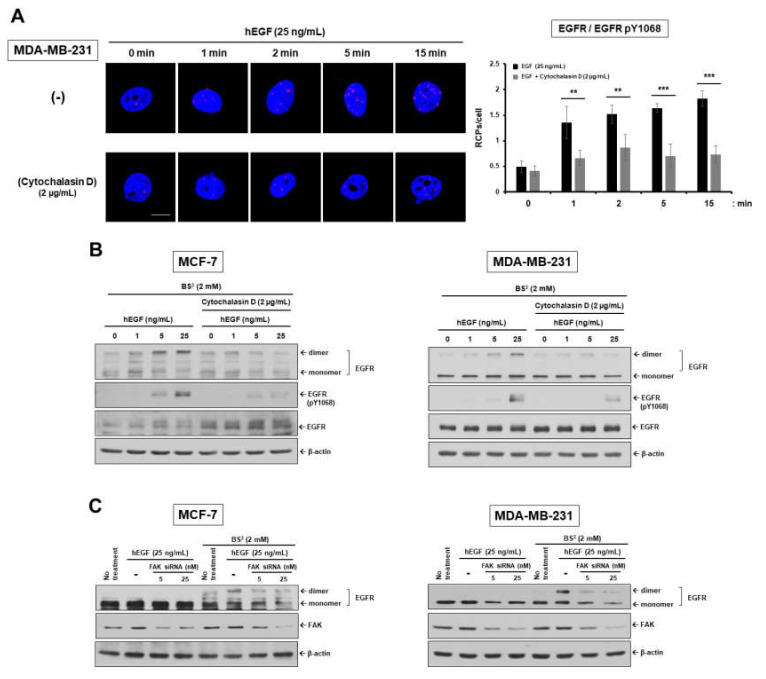

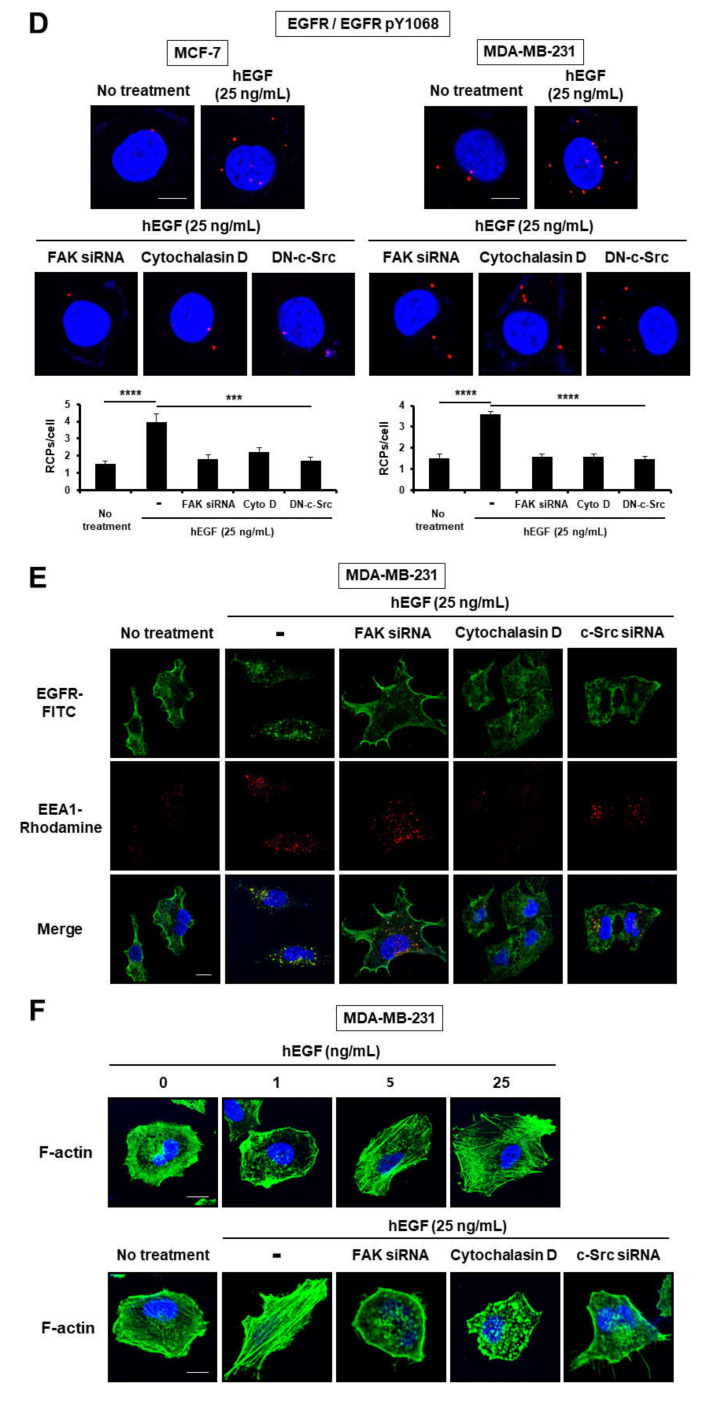
EGF induces EGFR dimerization and endocytosis through c-Src/FAK-mediated cytoskeleton reorganization. (**A**,**D**) The dimerization level of EGFR was assessed by in situ proximity ligation assay (PLA). (**B**,**C**) For dimerization assay, human breast carcinoma cells were treated with increasing concentrations of EGF in the presence or absence of cytochalasin D for 1 h on ice, to allow for EGF-induced EGFR dimerization but not endocytosis. Cells were subjected to BS^3^ chemical-mediated crosslinking as described in Materials and Methods. To determine the phosphorylation level of EGFR at Y1068, cells were incubated in serum-free medium (SFM) with EGF for 15 min at 37 °C. Cell extracts were assessed by Western blot analysis with the indicated antibodies. β-actin was used as a loading control. ** *p* < 0.01; *** *p* < 0.001; **** *p* < 0.0001. (**E**,**F**) EGFR endocytosis and actin cytoskeleton organization were determined by immunofluorescent assay (IFA). (**A**,**D**,**E**,**F**) Original magnification of representative images, 600×. Scale bars = 10 μm.

Previous studies showed that lovastatin, a statin medication, inhibits EGF-induced EGFR dimerization, activation, and downstream signaling through inhibition of RhoA-mediated actin polymerization and that ligand-induced remodeling of the actin cytoskeleton is required for clustering of transmembrane receptors, thereby resulting in endocytosis and signal transduction [26,27,28]. We determined whether impairing actin polymerization could disrupt ligand-induced dimerization of receptor tyrosine kinases in two human breast cancer cell lines, low EGFR-expressing MCF-7 and high EGFR-expressing MDA-MB-231. Recombinant human EGF induced EGFR dimerization in a time-dependent manner in MDA-MB-231 cells, but not in actin filament-disrupted cells treated with cytochalasin D (Figure 1A). EGF treatment could induce actin polymerization and stimulate EGFR dimerization and phosphorylation at tyrosine (Y) 1068 residue in a dose-dependent manner ( Figure 1B,F and Figure S1A,B,E). In contrast, disruption of actin filaments by cytochalasin D significantly interfered with EGF-induced phosphorylation and dimerization of EGFR. Dose-dependent inhibitory effect of cytochalasin D on EGFR dimerization was confirmed by in situ proximity ligation assay (PLA) (Figure S1B). Furthermore, we confirmed that actin polymerization is critical for not only EGFR/EGFR homo-dimerization but also EGFR/HER2 hetero-dimerization. EGFR/HER2 hetero-dimerization was observed from a very early time point after treatment of MCF-7 cells with EGF and increased in a time-dependent manner, whereas cotreatment with cytochalasin D significantly reduced hetero-dimerization (Figure S1C). These results indicate that actin polymerization is necessarily required for ligand-induced receptor dimerization.

Recruitment and activation of c-Src and FAK have been implicated in cell adhesion and motility by regulating actin cytoskeleton rearrangement and focal adhesion dynamics via activation of RhoA or Rac1/Cdc42 GTPases [29,30,31]. We determined whether inhibition of FAK function affects EGFR dimerization in the breast carcinoma cells. It was observed that EGF dose-dependently induced FAK phosphorylation at residue Y397 (Figure S1A). FAK knockdown revealed a markedly decreased rate of EGFR dimerization upon EGF binding (Figure 1C). To further investigate the functional relationship between c-Src/FAK-mediated actin rearrangement and EGFR dimerization and endocytosis, we carried out in situ PLA and immunofluorescent assay (IFA) after treatment with FAK small interfering RNA (siRNA), cytochalasin D, and dominant negative c-Src plasmid. Impairing actin polymerization with cytochalasin D or inhibiting c-Src/FAK signaling using dominant negative c-Src (DN-c-Src) or siRNA against FAK or c-Src inhibited EGF-induced EGFR receptor–receptor interaction, endocytosis, as well as actin polymerization (Figure 1D–F and Figure S1D,E). These results suggest that c-Src/FAK-mediated actin cytoskeleton rearrangement plays an important role in ligand-induced EGFR dimerization and activation.

### 2.2. EGF Induces EGFR Dimerization and Endocytosis through FAK-Mediated RhoA and Rac1 Signaling

**Figure 2:**
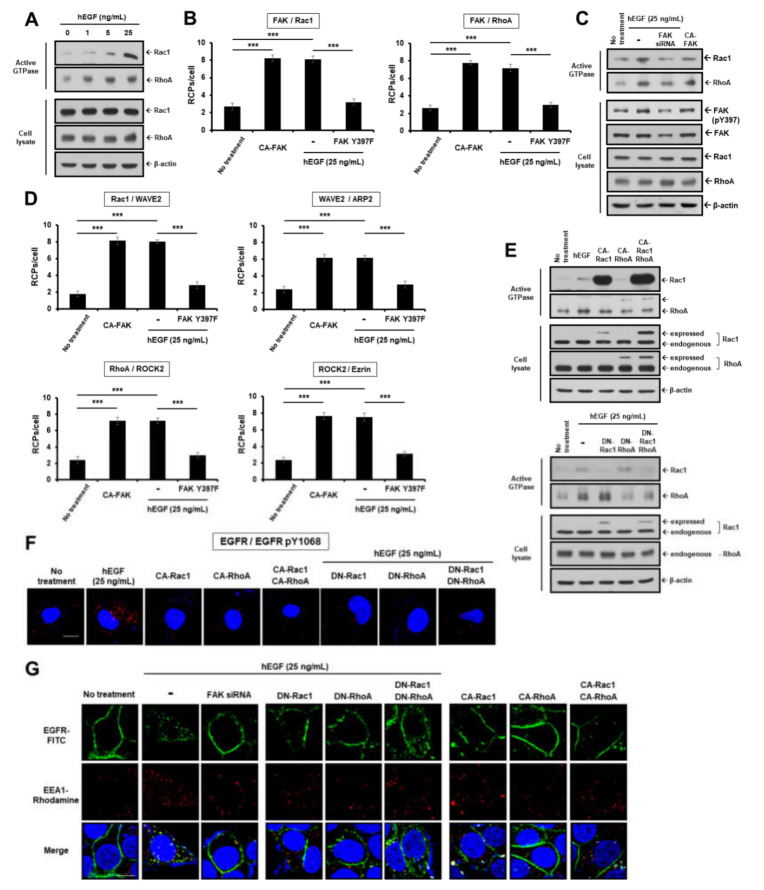
FAK functions as a critical mediator in EGF-induced activation of Rac1 and RhoA GTPases during EGFR signaling. (**A**,**C**) MCF-7 cells stimulated by binding of ligand to its receptor were analyzed for activation of small GTPases. Activated GTP-bound Rac1 or RhoA in the cell lysates were determined by immunoblotting with anti-Rac1 or anti-RhoA antibodies. β-actin was used as a loading control. (**B**,**D**) MDA-MB-231 cells were transfected with CA-FAK or FAK Y397F plasmids and incubated in the presence or absence of 25 ng/mL EGF at 37 °C, 5% CO_2_ for 15 min. The interactions between the pairs of molecules indicated were assessed by in situ PLA. *** *p* < 0.001. (**E**) Activation of small GTPases in MCF-7 cells was determined by immunoblotting. (**F**) EGFR dimerization in MDA-MB-231 cells was assessed by in situ PLA and the experiments were duplicated. (**G**) EGFR endocytosis in MCF-7 cells was determined by IFA as described above. Original magnification of representative images, 600×. Scale bars = 10 μm.

Actin cytoskeletal reorganization is regulated by the Rho family of GTPases, including Rho, Rac, and CDC42 [32,33,34,35]. We found that although MCF-7 has low expression level of EGFR, EGF treatment dose-dependently stimulates upregulation of the activity of GTPases, Rac1 and RhoA, which is consistent with the results in Figure 1F and Figure S1E showing the pattern of increase in F-actin polymerization (Figure 2A). To determine the role of FAK in activating small GTPase signaling, we transiently transduced constitutively active FAK mutant (CA-FAK), dominant-negative FAK mutant (FAK Y397F) or FAK siRNA. Interaction of FAK with both the GTP-binding proteins and their GTPase activities were upregulated by overexpressing CA-FAK or treating with EGF (Figure 2B,C and Figure S2A). Contrarily, the increased interaction of GTPases with FAK and their upregulated GTPase activities were suppressed by overexpression of kinase-dead FAK Y397 mutant or by knockdown of FAK using siRNA. In addition, knockdown of FAK resulted in inhibition of EGF-induced EGFR endocytosis (Figure 2G). Furthermore, interactions among signaling molecules downstream of GTPases, including Wiskott-Aldrich syndrome protein (WASp) family Verprolin-homologous protein-2 (WAVE2), Actin-related protein-2 (ARP2), ROCK2, and Ezrin, showed patterns similar to those of FAK with RhoA and Rac1 (Figure 2D and Figure S2B). These results show that FAK contributes as a key regulator of RhoA and Rac1, leading to activation of GTPase signaling.

**Figure 3:**
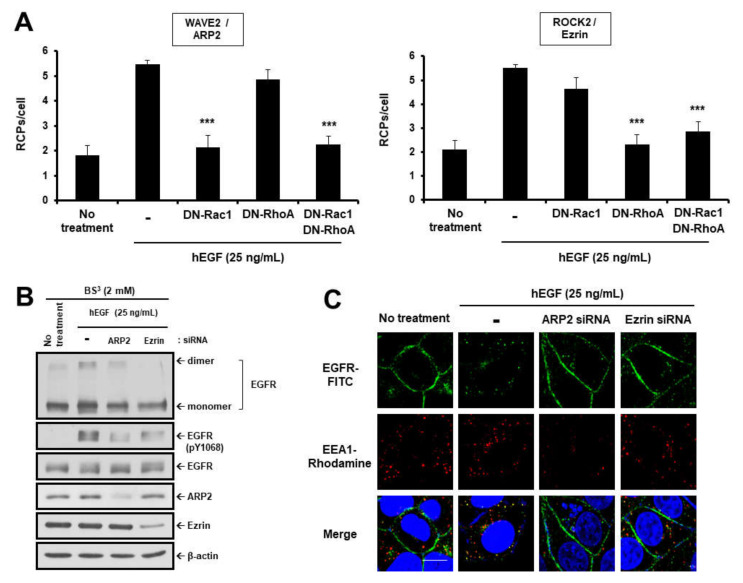

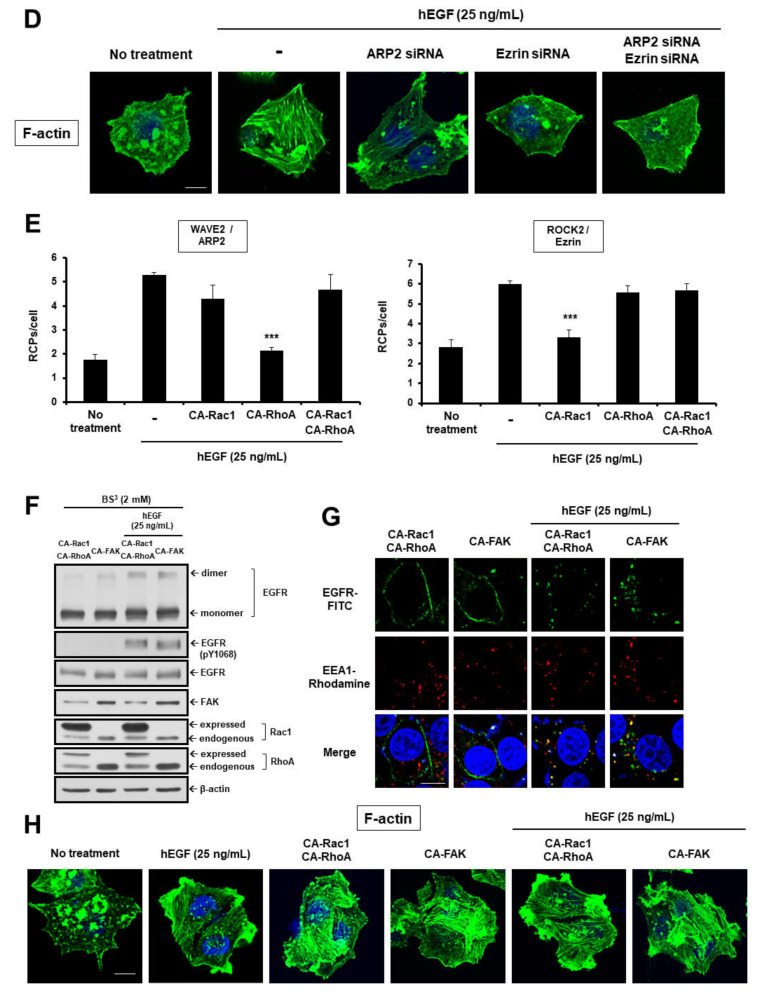
Modulation of actin polymerization by Rac1/RhoA GTPases is essential for EGF-induced dimerization and endocytosis of EGFR. (**A**,**E**) The changes in the activation of Rac1/RhoA-mediated signaling were observed in MCF-7 cells. The interactions between the pairs of molecules indicated were assessed by in situ PLA. *** *p* < 0.001. (**B**,**F**) To determine EGFR dimerization, MDA-MB-231 cells were subjected to BS^3^ chemical-mediated crosslinking, as described above and in the Materials and Methods. Cell extracts were assessed via Western blotting to determine the dimerization and phosphorylation levels of EGFR and the expression levels of indicated proteins. β-actin was used as a loading control. EGFR endocytosis (**C**,**G**) and actin cytoskeleton organization (**D**,**H**) in MCF-7 cells transfected with siRNAs specific for ARP2 and Ezrin or plasmids encoding CA-GTPases or CA-FAK. Original magnification of representative images, 600×. Scale bars = 10 μm.

Next, we investigated the effects of activating and inhibiting RhoA and Rac1 GTPases on dimerization and endocytosis of EGFR. Transiently transfected MCF-7 cells expressing CA-Rac1 or CA-RhoA showed significantly enhanced GTPase activity upon EGF treatment (Figure 2E). However, the CA-GTPases influenced neither the dimerization of EGFR nor its endocytosis, even though they induced actin cytoskeleton polymerization (Figure 2F,G, Figure 3F and Figure S2C). On the other hand, DN-Rac1 or DN-RhoA specifically inhibited EGF-stimulated activation of these GTPases (Figure 2E). Contrary to the effect of CA-GTPases, DN-GTPases efficiently suppressed both EGFR dimerization and endocytosis, which were induced by EGF (Figure 2F,G and Figure S2C). We further confirmed the effect of GTPase signaling activity on EGFR dimerization and endocytosis. Consistent with the results in Figure 2E, transfection with DN-Rac1 specifically inhibited the interaction between WAVE2 and ARP2, while transfection with DN-RhoA inhibited only ROCK2–Ezrin interaction, but not WAVE2–ARP2 interaction (Figure 3A). However, EGF-induced dimerization and phosphorylation at Y1068 of EGFR in MDA-MB-231 cells were significantly reduced, even by a single knockdown of ARP2 or Ezrin (Figure 3B). In ARP2 knockdown cells, EGF-induced EGFR endocytosis as well as actin filament branching was significantly inhibited (Figure 3C,D). Knockdown of Ezrin disrupted actin filament polymerization and also suppressed endocytosis of EGFR. In addition, simultaneous knockdown of ARP2 and Ezrin also inhibited actin polymerization. Consistent with the results of DN-GTPases, transfection with CA-Rac1 stimulated the interaction between WAVE2 and ARP2, whereas transfection with CA-RhoA stimulated the interaction of ROCK2 with Ezrin (Figure 3E). Constitutively active forms of Rac1, RhoA, or FAK induced actin filament polymerization in the presence or absence of EGF (Figure 3H). However, although they induced actin filament polymerization as efficiently as EGF, CA-Rac1, CA-RhoA, or CA-FAK failed to stimulate EGFR dimerization and endocytosis without EGF treatment (Figure 3F,G). In addition, EGF binding to EGFR is necessary to initiate the phosphorylation of EGFR, regardless of actin polymerization. These results suggest that GTPase-driven actin polymerization is necessary, but not sufficient for EGFR dimerization and endocytosis.

### 2.3. CD99 Activation Attenuates EGF-Induced Dimerization and Activation of EGFR via the PKA/SHP2/HRAS/PTPN12/FAK Signaling Pathway

**Figure 4:**
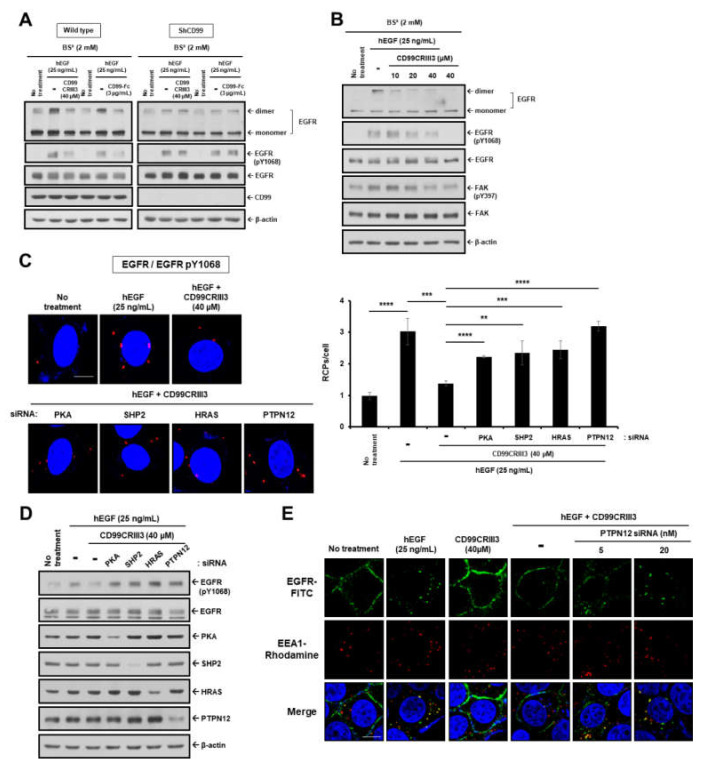
CD99 agonistic ligands inhibit EGFR dimerization and endocytosis via the PKA/SHP2/HRAS/ERK/PTPN12 signaling pathway. (**A**,**B**) For dimerization assay, wild type or CD99 shRNA stable-expressing MDA-MB-231 cells were treated with CD99CRIII3 or CD99-Fc in the presence of EGF (25 ng/mL) for 1 h on ice. Cells were subjected to BS^3^ chemical-mediated crosslinking. Cell extracts were subjected to SDS-PAGE to assess EGFR dimerization and phosphorylation levels of EGFR at Y1068 and FAK at Y397 and the expression levels of indicated proteins. β-actin was used as a loading control. (**C**) In MCF-7 cells, dimerization of EGFR was assessed by in situ PLA. ** *p* < 0.01; *** *p* < 0.001; **** *p* < 0.0001. (**D**) Whole cell lysates extracted from MDA-MB-231 cells treated with EGF with or without CD99CRIII3 were subjected to SDS-PAGE to examine the phosphorylation levels of EGFR and expression levels of each target protein. (**E**) EGFR endocytosis in MCF-7 cells treated with EGF or CD99CRIII3 either alone or combined. (**C**,**E**) Original magnification of representative images, 600×. Scale bars = 10 μm.

To determine whether CD99 activation can disrupt EGF-induced dimerization and activation of EGFR, two breast cancer cell lines, MDA-MB-231 and MCF-7, were treated with CD99 agonist ligands, CD99CRIII3 or CD99-Fc. We previously demonstrated that CD99CRIII3, a CD99-derived peptide, can function as a CD99 agonist as efficiently as CD99 protein derivatives or anti-CD99 agonist monoclonal antibody [24]. Western blotting and in situ PLA showed that those two CD99 agonists significantly inhibited EGFR dimerization and phosphorylation at Y1068, which were induced by EGF (Figure 4A and Figure S4A). Moreover, neither CD99-Fc nor CD99CRIII3 had any effect on the EGF-induced dimerization and phosphorylation of EGFR at Y1068 in CD99-knockdown cells. EGFR dimerization and phosphorylation at Y1068 were significantly reduced by CD99CRIII3 in a dose-dependent manner (Figure 4B and Figure S4B). CD99CRIII3 inhibited EGF-induced phosphorylation of FAK at Y397. Consistent with this, CD99-Fc and CD99CRIII3 coordinately inhibited EGF-induced interactions of FAK with Rac1 and RhoA in MCF-cells, but not in CD99-knockdown MCF-7 cells (Figure S4C,D). In addition, both molecules suppressed the EGF-induced actin organization in a dose-dependent manner (Figure S4E). These results demonstrate that CD99 agonists inhibited EGF-induced dimerization and activation of EGFR by disrupting FAK-mediated actin organization.

Since our previous results showed that CD99CRIII3 dephosphorylated FAK at Y397 through the PKA/SHP2/HRAS/PTPN12 signaling pathway [24], we next determined whether CD99CRIII3 regulates EGF-induced dimerization and activation of EGFR via the PKA/SHP2/HRAS/PTPN12 signaling pathway. Knockdown of PKA, SHP2, HRAS, or PTPN12 restored EGFR dimerization, which had been inhibited by treatment with CD99CRIII3 (Figure 4C). CD99CRIII3-mediated dephosphorylation of EGFR at Y1068 was also inhibited by knockdown of each of the intracellular signaling molecules (Figure 4D). In particular, knockdown of PTPN12 abrogated the inhibitory effect of CD99CRIII3 on EGFR endocytosis induced by EGF (Figure 4E). Consistent with our previous results, CD99CRIII3 efficiently inhibited EGF-induced actin rearrangement and EGFR dimerization by dephosphorylating FAK at Y397 via the PKA/SHP2/HRAS/PTPN12 signaling pathway.

**Figure 5:**
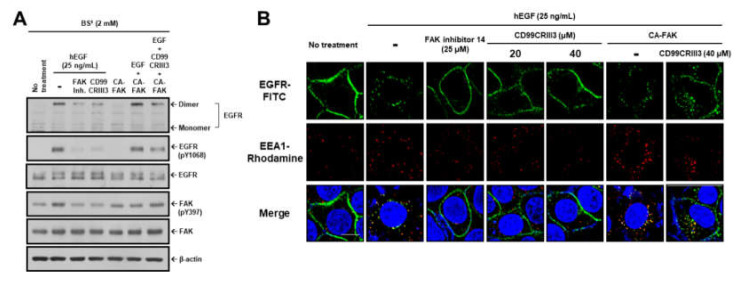

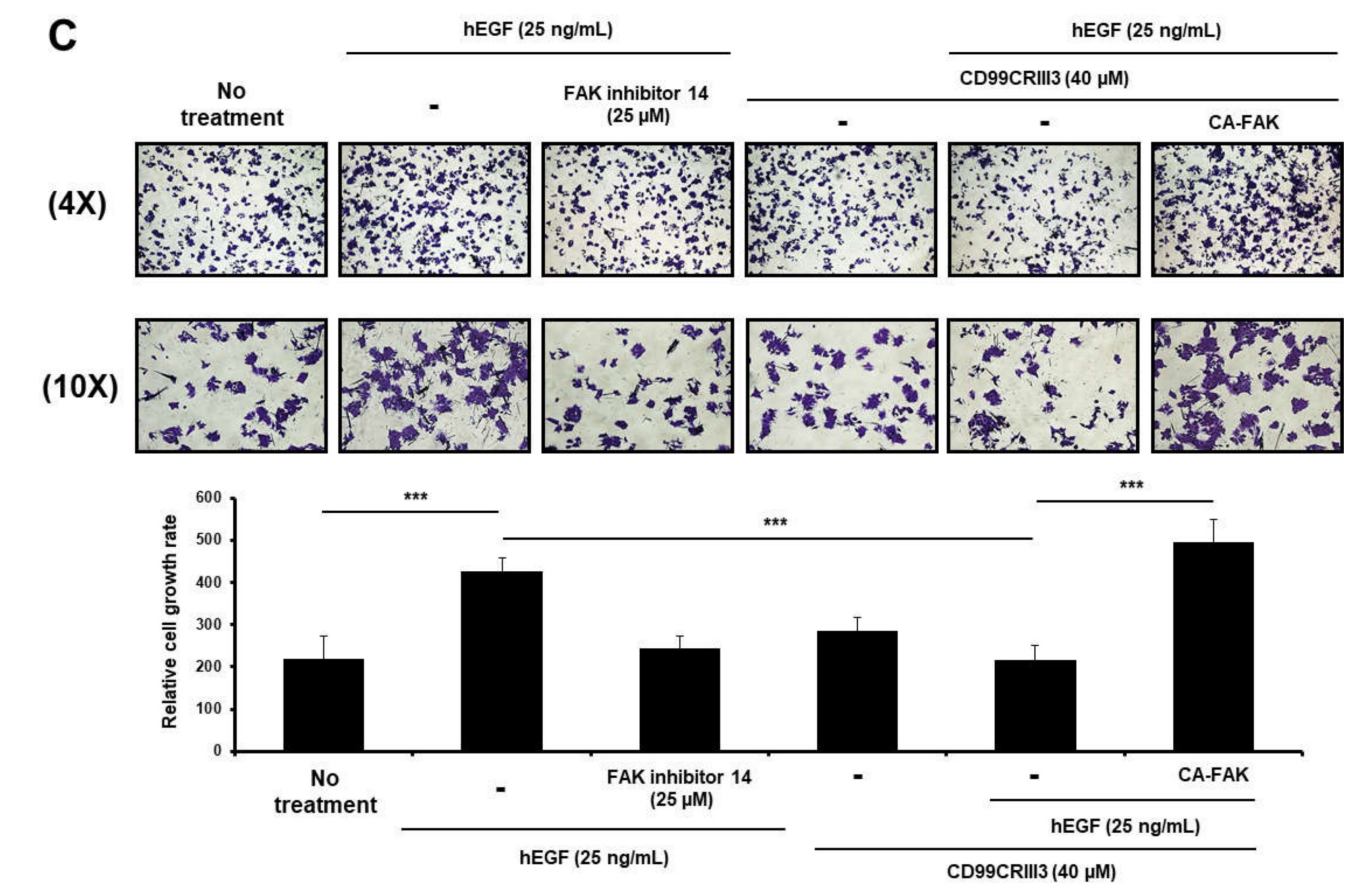
FAK plays an important role in breast cancer cell proliferation induced by EGFR activation. (**A**) MDA-MB-231 cells transfected with CA-FAK plasmid were subjected to BS^3^ chemical-mediated crosslinking as described above. Cell extracts were assessed by Western blot analysis to determine the dimerization and phosphorylation levels of EGFR or the expression and phosphorylation levels of FAK. β-actin was used as a loading control. (**B**) EGFR endocytosis in MCF-7 cells was assessed by IFA as described above. Original magnification of representative images, 600x. Scale bars = 10 μm. (**C**) MCF-7 cells were stained with crystal violet. Images were captured using a Nikon Eclipse TE2000-U and the representative images are shown. Lines indicate statistical comparisons, and significant differences between treatments are shown by asterisks as follows: *** *p* < 0.001.

To further validate the function of CD99CRIII3 in regulating FAK activity, the cells were treated with a selective FAK inhibitor 14 or transfected with CA-FAK. Inhibition of FAK activity by FAK inhibitor 14 attenuated EGF-induced EGFR dimerization, phosphorylation, and endocytosis by a similar degree to that attenuated by CD99CRIII3 treatment (Figure 5A,B). In contrast, CA-FAK partially recovered the functional activities of EGFR, which had been suppressed by CD99CRIII3, suggesting that persistent activation of FAK partially resists the inhibitory effects of CD99CRIII3 on EGFR dimerization, phosphorylation, and endocytosis. However, CA-FAK alone did not have any effect on dimerization and endocytosis of EGFR. EGFR regulates various cellular signals related to cell growth, proliferation, differentiation, and tumorigenesis [36,37,38]. As expected, EGF-induced activation of EGFR significantly increased proliferation of MCF-7 cells, whereas CD99CRIII3- or FAK inhibitor 14-induced inhibition of EGFR resulted in a reduced proliferation rate (Figure 5C). The cell proliferation rate, which was suppressed by CD99CRIII3 treatment, was completely restored in both types of cells when transfected with CA-FAK. These results demonstrate that CD99 agonist ligands suppress EGF-induced activation of EGFR through the PKA/SHP2/HRAS/ERK/PTPN12/FAK signaling pathway.

### 2.4. CD99CRIII3-Activated PTPN12 Regulates the Activation of EGFR Signaling through the PTPN12/FAK/ Rho/Rac Axis

**Figure 6:**
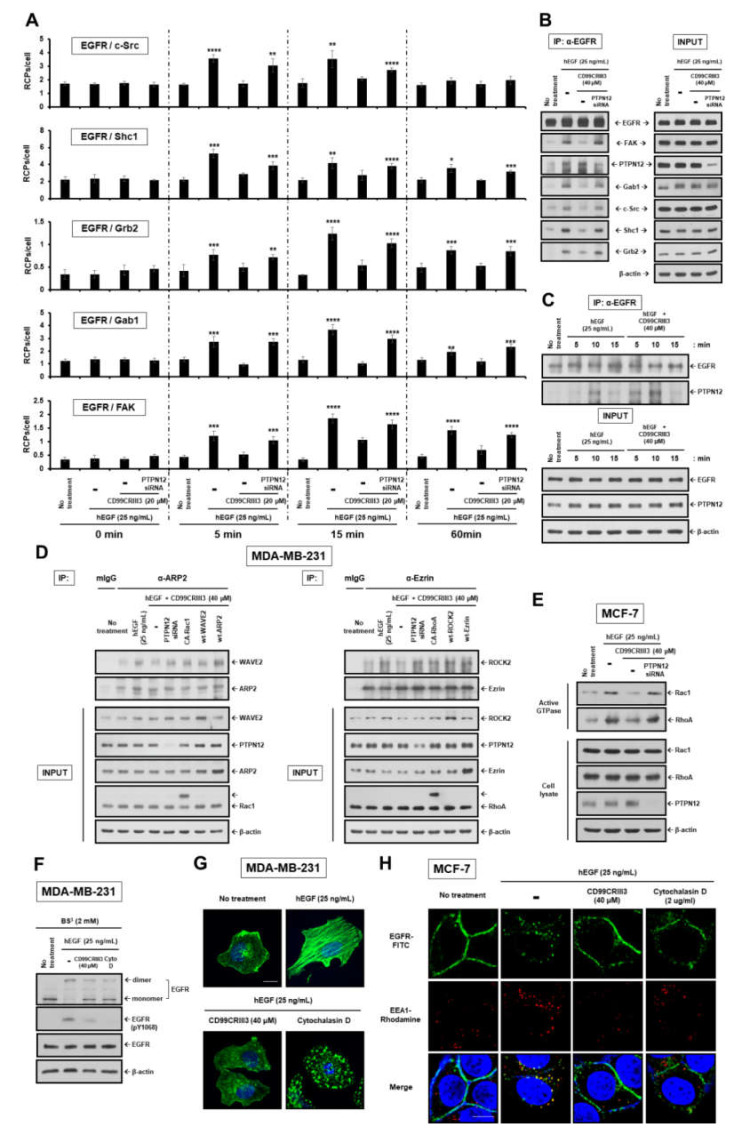
CD99CRIII3 activates PTPN12 to facilitate inhibition of EGFR signaling. (**A**) In situ PLA performed to assess the interactions between the pairs of molecules indicated in MCF-7 cells. * *p* < 0.05; ** *p* < 0.01; *** *p* < 0.001; **** *p* < 0.0001. (**B**) MDA-MB-231 cells were transfected with PTPN12 siRNA, followed by treatment with EGF (25 ng/mL) with or without CD99CRIII3 (40 µM) for 15 min. (**C**) MDA-MB-231 cells were treated with EGF and/or CD99CRIII3 in a time-dependent manner. (**D**) MDA-MB-231 cells were transiently transfected with PTPN12 siRNA or expression plasmids encoding CA-GTPases or WAVE2, ARP2, ROCK2, and Ezrin. Cell lysates were immunoprecipitated with the antibodies indicated. The immunoprecipitates were analyzed by Western blot with the antibodies indicated. (**E**) The cells stimulated by binding of ligand to its receptor were assayed for activation of small GTPases. β-actin was used as a loading control. (**F**) For dimerization assay, MDA-MB-231 cells were subjected to BS^3^ chemical-mediated crosslinking as described above. Cell extracts were assessed by Western blot analysis to determine the dimerization and phosphorylation levels of EGFR. β-actin was used as a loading control. Actin cytoskeleton organization in MDA-MB-231 cells (**G**) and EGFR endocytosis in MCF-7 cells (**H**) were determined by IFA as described above. Original magnification of representative images, 600×. Scale bars = 10 μm.

As described above, PTPN12 acts as a negative regulator of multiple RTKs implicated in tumor progression [8,13,15,39]. We hypothesized that PTPN12 may restrain the activation of several cytoplasmic adaptor and kinase proteins that are recruited to EGFR following ligand binding, resulting in attenuation of the activated intracellular signals. Here, using in situ PLA assay, we evaluated the effect of CD99CRIII3-induced PTPN12 on EGFR signaling cascade. Consistent with our hypothesis, EGF treatment facilitated the interactions of EGFR with c-Src, Shc1, Grb2, Gab1, and FAK (Figure 6A). In particular, EGFR showed strongest interaction with c-Src and Shc1 after 5 min of treatment with EGF, whereas the highest degree of interaction between Grb2, Gab1, FAK, and EGFR was observed after 15 min of treatment. Surprisingly, CD99CRIII3 completely attenuated all interactions induced by EGF binding, whereas knockdown of PTPN12 caused a restoration of EGF-induced interactions between EGFR and other intracellular proteins. Consistent with these findings, co-immunoprecipitation revealed that EGF-induced interactions of EGFR with the intracellular molecules were attenuated by co-treating with CD99CRIII3. However, CD99CRIII3 lost its inhibitory effect on EGF-induced EGFR activation by knockdown of PTPN12 (Figure 6B and Figure S5A). To further characterize the kinetics of EGFR-PTPN12 interaction, we evaluated the time-dependent pattern of EGFR-PTPN12 interaction following stimulation with EGF alone or combined stimulation with EGF and CD99CRIII3. PTPN12 was found to co-precipitate with EGFR after treatment with EGF. After 10 min of treatment it showed the highest degree of interaction with EGFR (Figure 6C and Figure S5B). In contrast, the interaction between both molecules in cells treated with EGF plus CD99CRIII3 occurred much earlier than that in cells treated with EGF only and was continued until 10 min after treatment. These results show that PTPN12 activated by CD99CRIII3 plays a critical role in the disruption of the intracellular adapter/kinase complex involved in the EGFR signaling cascade.

It is certain that PTPN12 is involved in regulating cellular motility and morphology, since the phosphatase acts as a central regulator of actin cytoskeleton reorganization [8,9,40]. We performed co-immunoprecipitation to further verify the effects of CD99CRIII3-activated PTPN12 on Rac1/RhoA GTPase signaling pathways. CD99CRIII3 strongly inhibited WAVE2–ARP2 and ROCK2–Ezrin interactions, which were stimulated by EGF treatment in MDA-MB-231 cells (Figure 6D). Contrarily, knockdown of PTPN12 was able to neutralize the effects of CD99CRIII3 and maintain EGF-induced interactions between WAVE2 and ARP2 as well as ROCK2 and Ezrin. Consistent with these observations, while CD99CRIII3 suppressed the EGF-induced activation of Rac1 and RhoA GTPases, its inhibitory effect was neutralized by siRNA-mediated knockdown of PTPN12 in MCF-7 cells (Figure 6E). In addition, transfection with plasmids encoding CA-Rac1, wt-WAVE2, or wt-ARP2 reinstated the Rac1-mediated interaction of WAVE2 with ARP2, which had been inhibited by CD99CRIII3. Similar results were obtained in RhoA-mediated signaling cascade by expression of CA-RhoA, wt-ROCK2, or Ezrin. These results were demonstrated by in situ PLA and immunocytochemical assay for monitoring the localization of each of the Rac1/RhoA GTPase signaling-related proteins and the interactions between them (Figure S6A,B). CD99CRIII3 inhibited colocalization and physical proximity of WAVE2 and ARP2, ROCK2 and Ezrin at the cell membrane region, which were induced by treatment with EGF. Consistent with the above results, knockdown of PTPN12 abrogated the inhibitory effect of CD99CRIII3 on the interactions of actin polymerization-regulating proteins. Moreover, transfection with CA-Rac1 or overexpression of either of WAVE2 or ARP2 maintained the interaction between WAVE2 and ARP2. We also identified similar patterns of results showing RhoA-dependent localization of ROCK2 and Ezrin. Taken together, we found that CD99CRIII3 inhibits EGF-induced dimerization and endocytosis of EGFR, as well as actin polymerization in a PTPN12-dependent manner at a level equivalent to that exhibited by cytochalasin D treatment (Figure 6F–H and Figure S6C). These observations suggest that PTPN12 functions as a key regulator in CD99CRIII3-induced inhibition of EGFR dimerization and endocytosis via suppression of actin polymerization.

### 2.5. CD99CRIII3 Dose-Dependently Inhibited TNBC Progression In Vivo through PTPN12-Mediated Suppression of Breast Cancer Cell Proliferation

**Figure 7:**
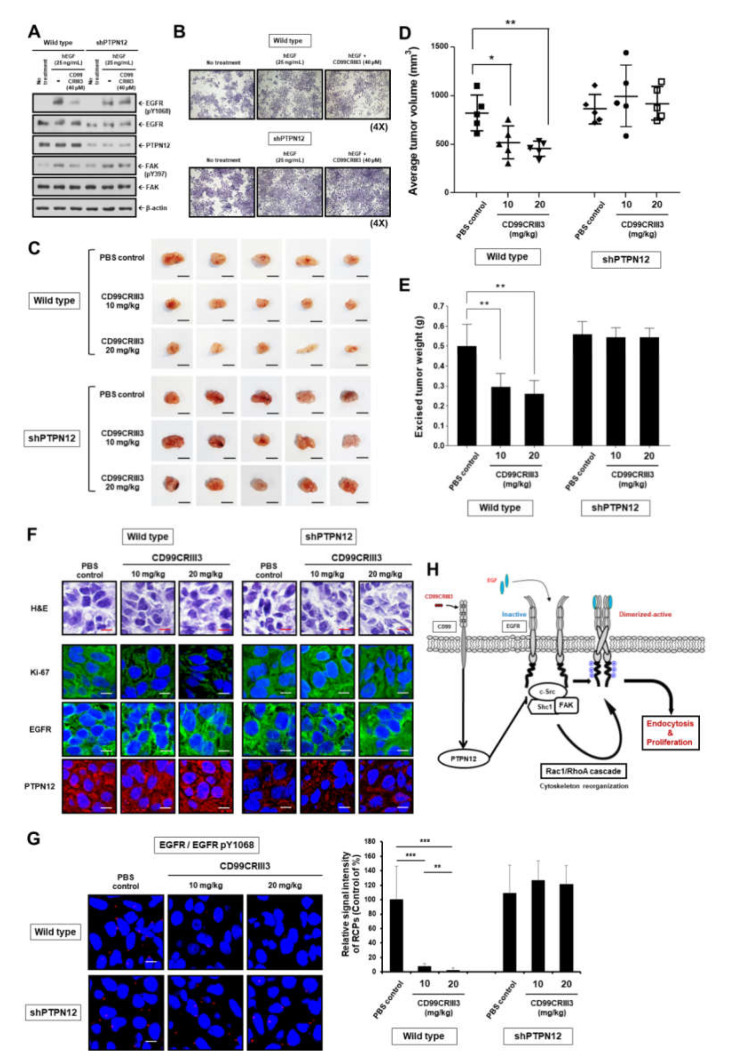
Evaluation of the in vivo efficacy of CD99CRIII3 in the xenograft model of human breast cancer. (**A**) MDA-MB-231 cells were transiently transfected with PTPN12 shRNA plasmid. Cell extracts were assessed by Western blot analysis to determine the phosphorylation levels of EGFR at Y1068 or FAK at Y397. β-actin was used as a loading control. The uncropped Western blots have been shown in Figure S8. (**B**) Comparison of cell growth rate between wt-MDA-MB-231 and shPTPN12-MDA-MB-231 cells. (**C**) Images of tumor xenografts. Scale bar represents 10 mm. (**D**,**E**) Graph shows the mean difference in tumor volume and weight between wild-type and PTPN12 knockdown cells. Lines indicate statistical comparisons, and significant differences between treatments are shown by asterisks as follows: * *p* < 0.05; ** *p* < 0.01. (**F**) Hematoxylin and Eosin (H&E) staining of tumor xenografts. Scale bar represents 10 μm for 400× magnification. The expression levels of EGFR, PTPN12, and Ki67 by IHC in xenografts. (**G**) In situ PLA analysis was performed to determine the dimerization pattern of EGFR within xenograft tumor tissues. Confocal images were taken from four tumors of each treatment group and displayed in compressed z-stack form. Numerical values are the mean intensities (±SD) of red spots in three randomly selected fields per tumor section. Significant differences between treatments are shown by asterisks as follows: ** *p* < 0.01; *** *p* < 0.001. Scale bar, 10 μm (600×). wt, wild type; sh, short hairpin. (**H**) Schematic model for the inhibitory effect of CD99 agonistic ligand on EGF-induced activation of EGFR.

To determine the anti-tumorigenic effect of CD99CRIII3 and the importance of PTPN12 in suppressing tumor progression, we generated a stable MDA-MB-231 cell line (shPTPN12-MDA-MB-231) with 75% reduction in PTPN12 protein using the shRNA system (Figure S7A). shPTPN12-MDA-MB-231 cells exhibited no changes in the expression level of EGFR and CD99. In addition, the dimerization and phosphorylation on Y1068 of EGFR and Y397 of FAK, which had been suppressed by CD99CRIII3 in wt-MDA-MB-231 cells, were not affected in shPTPN12-MDA-MB-231 cells (Figure 7A and Figure S7B). CD99CRIII3 inhibited the proliferation of wt-MDA-MB-231 cells, which was increased by treatment with EGF. Contrarily, the cell proliferation rate, which was enhanced by EGF, was not suppressed by CD99CRIII3 in the PTPN12 knockdown cell line (Figure 7B and Figure S7C). We confirmed the physiological characteristics of shPTPN12-MDA-MB-231 using in situ PLA (Figure S7D). EGF treatment induced the interactions of EGFR with Grb2, c-Src, Shc1, FAK, Gab1, and itself. However, the shPTPN12-MDA-MB-231 cells did not inhibit these interactions by treatment with CD99CRIII3. These results suggest that CD99CRIII3 inhibits EGF-induced EGFR activation via PTPN12-mediated signaling.

Finally, we carried out a tumor xenograft assay in the BALB/c nude mouse model with wt- and shPTPN12-MDA-MB-231 human breast carcinoma cells. The daily injection of CD99CRIII3 led to significantly decreased tumor volume and weight in the wt-MDA-MB-231-inoculated mice, compared with PBS-treated mice (Figure 7C–E). However, there were no differences in tumor size and weight in the CD99CRIII3-treated mice injected with shPTPN12-MDA-MB-231 cells, indicating that CD99CRIII3 exerts its anti-tumorigenic activity via the CD99–PTPN12 axis. After 15 days of CD99CRIII3 administration, tumors were collected and sliced into small pieces. Serial sections of the tumor specimens were stained with hematoxylin and eosin (H&E) and antibodies to measure the expression of Ki67, EGFR, and PTPN12 (Figure 7F). Histological analysis of the specimens revealed that CD99CRIII3 led to reduced Ki-67 expression in a group of wt-MDA-MB-231-inoculated mice, but not in the shPTPN12-MDA-MB-231-inoculated mice group. The majority of tumor mass was identified to be positive for EGFR and Ki-67. The number of Ki-67-positive cells was reduced in the CD99CRIII3-treated mice, showing correlation with the reduced tumor size (Figure S7E). In addition, CD99CRIII3 dose-dependently inhibited EGFR dimerization only in wt-MDA-MB-231-originated tumor tissues (Figure 7G). These results indicate that CD99 agonist ligand could significantly suppress the proliferation of MDA-MB-231 human breast cancer cells through PTPN12-mediated inactivation of EGFR.

## 3. Discussion

In this study, we clearly showed that actin polymerization plays an important role in EGFR receptor dimerization and activation, which was inhibited by the CD99/PTPN12/FAK/Rho/Rac axis. Our novel findings provide an insight into the role of CD99 in the inactivation of several oncogenic tyrosine kinases including EGFR [13,14].

As the actin cytoskeleton plays an important role in controlling the movement of intracellular organelles as well as cell surface receptors [41,42,43], we elucidated the importance of cytoskeleton reorganization in ligand-induced dimerization and subsequent activation of EGFR. EGFR directly associates with the actin filament via its C-terminal actin-binding domain [44,45]. We found that impairment of actin cytoskeleton organization by cytochalasin D inhibits the phosphorylation, dimerization, and internalization of EGFR, consistent with previous results showing that disruption of actin polymerization inhibits ligand-induced EGFR dimerization, activation, and downstream signaling [26,28]. Besides the role for EGFR dimerization, the blocking of F-actin polymerization inhibits the CXCL12-mediated dimerization of CXCR4 [46]. The actin cytoskeleton intimately interacts with plasma membrane integral proteins and regulates intricate membrane events, such as the formation of focal adhesions as well as the internalization, recycling, compartmentalization, dynamics, clustering, and diffusion of membrane receptor proteins [27,41,47]. The assembly and disassembly of cytoskeletal actin filaments (F-actin) are regulated by c-Src and FAK [31,48,49]. Functional impairment of c-Src or FAK inhibited actin polymerization, leading to the suppression of dimerization and internalization of EGFR. In contrast, transduction with CA-FAK facilitated the FAK-mediated activation of Rac1/RhoA signaling pathways and actin polymerization. Importantly, it looks like dimerization of EGFR is not sufficient to activate its kinase activity. Although CA-FAK could induce the formation of actin filaments without EGF ligand, it failed to proceed to the next step, EGFR dimerization and endocytosis. CA-Rac1 and CA-RhoA also showed similar results. The binding of growth factor ligands to EGFR may be critical for conformational changes of the receptor or its dynamics, leading to dimerization or oligomerization of EGFR and subsequent activation of the downstream signaling pathway. In other words, EGFR activation may require both ligand binding to EGFR and subsequent dimerization. Therefore, disruption of ligand-induced EGFR dimerization as well as ligand binding would be a promising therapeutic strategy for the treatment of breast cancer patients with aberrant expression or activation of EGFR.

Breast cancer can be classified into several subtypes according to the expression level of various surface marker proteins, including estrogen receptor (ER), progesterone (PR), and HER2. On the other hand, EGFR is expressed in a wide range of breast cancer cell lines at different levels. EGFR has long been in spotlight as a reasonable target molecule for developing antitumor strategies, since its aberrant activation by increased expression of a constitutively activated truncated variant EGFRvIII or itself is implicated in the development and progression of a broad range of solid cancer diseases including breast cancer [50]. We adopted two breast cancer cell lines, MDA-MB-231 and MCF-7. MDA-MB-231 cells lack the expression of ER, PR, and HER2, while they show high expression of multiple RTKs. On the other hand, adenocarcinoma MCF-7 cells express ER, PR, and glucocorticoid receptors. We found that MDA-MB-231 cells express EGFR at high levels, whereas MCF-7 cells express very low levels of this receptor. The expression levels of CD99, in contrast, are similar in both cell lines. Despite different levels of EGFR expression, these two breast carcinomas were similarly affected by EGFR ligands and CD99 agonists. These results suggest that EGFR might play a dominant role in cellular and physiological systems of breast cancer cells, so that it can be a valuable target for the development of a broadly applicable anti-cancer drug.

PTPN12 is a tumor suppressor which regulates cellular transformation from normal to malignant cells via the inhibition of multiple oncogenic tyrosine kinases [13,14]. Consistent with this, our data showed that stable knockdown of PTPN12 increased tumor progression in vivo. Although several studies imply the functional significance of PTPN12 in controlling tumor progression, the activator of PTPN12 has not been identified yet. One novel finding of this study is that the CD99–PTPN12 axis participates in the regulation of ligand-induced activation of EGFR by suppressing the reorganization of the actin cytoskeleton. This observation is consistent with a previous study, which demonstrated that PTPN12 controls EphA3 activation by regulating actin cytoskeletal organization during Eph clustering [8]. Furthermore, we previously reported the molecular mechanism by which CD99 induces β1 integrin inactivation via PTPN12 activation [24]. Likewise, CD99CRIII3 showed significant inhibitory effects on EGF-induced EGFR dimerization and internalization via activation of PTPN12. When EGFR is activated with its ligand, PTPN12 is recruited to the activated EGFR to return to an inactive state within 15 min [39]. On the other hand, CD99CRIII3 induced very early recruitment of PTPN12 to the EGF-induced EGFR signaling complex, which led to the inhibition of EGFR dimerization and activation. The remarkable inhibitory effect of CD99CRIII3 on EGFR activation was suppressed when CD99 or PTPN12 expression was downregulated, indicating that CD99 activation by CD99CRIII3 stimulated PTPN12 to inhibit the early stage of the EGFR signaling pathway.

PTPN12 exhibited relatively low expression levels in triple-negative breast cancer (TNBC) cells [13]. However, ectopic restoration of PTPN12 in TNBC resulted in the suppression of anchorage-independent proliferation and metastatic ability. Consistent with this finding, we observed that CD99CRIII3 significantly suppressed the EGF-induced proliferation of MDA-MB-231 and MCF-7 breast cancer cells, which was not observed in PTPN12-knockdown cells. Furthermore, shPTPN12-MDA-MB-231 cells allowed us to examine whether CD99CRIII3 affects in vivo tumorigenesis via the PTPN12-dependent negative feedback loop. CD99CRIII3 dose-dependently suppressed the growth of MDA-MB-231 human breast cancer cells implanted in nude mice, while it failed to suppress the growth of shPTPN12-MDA-MB-231 cells implanted in nude mice, suggesting that PTPN12 serves as a key executor of the CD99 signaling pathway. Consistently, CD99CRIII3 inhibited EGFR dimerization in wt-MDA-MB-231-originated tumor tissues, but not in shPTPN12-MDA-MB-231-originated tumor tissues. Here we pay attention to recent reports showing that CD99 activates p53 tumor suppressor by inducing degradation of Mdm2, an E3 ubiquitin ligase, resulting in the death of Ewing sarcoma (EWS) [20,51]. Collectively, these results suggest that CD99 might play a key role in modulating the activities of intracellular tumor suppressors, PTPN12 and p53, whose interrelationship still remains elusive.

The growth of various tumors is promoted by tumorigenic growth factor receptors, such as FGFR, TGF-βR, IGF-1R, InsR, and PDGF [52,53,54,55,56]. Given that their kinase activity is induced via dimerization and activation according to ligand binding, and actin cytoskeleton is implicated in controlling receptor compartmentalization [27,57,58], it is important to determine whether CD99CRIII3 can regulate the activity of those RTKs. Additionally, protein tyrosine phosphatases (PTPs) act as inhibitors, regulating tumor-inducing activity [53]. Thus, our results suggest that PTPN12 activated by CD99CRIII3 may suppress the activity of other abnormal RTKs as well as EGFR and that their dimerization and activation processes are regulated by actin cytoskeleton-controlled clustering. However, the underlying mechanism by which CD99CRIII3 inhibits the dimerization and activation of other tumorigenic RTKs needs further investigation in a broad range of tumors.

## 4. Materials and Methods

### 4.1. Reagents and Antibodies

All cultureware and reagents were purchased from Invitrogen (Carlsbad, CA, USA). Immun-Blot polyvinylidene fluoride (PVDF) membranes for protein blotting were purchased from Bio-Rad Laboratories (Hercules, CA, USA). The WEST-ZOL plus Western blot detection kit was obtained from iNtRON Biotechnology, Inc. (Seongnam, Korea). Lipofectamine LTX/PLUS and RNAiMAX reagents were purchased from Invitrogen (Life Technologies, Grand Island, NY, USA). Protein A/G agarose beads were purchased from Santa Cruz Biotechnology, Inc. (Santa Cruz, CA, USA). Cytochalasin D, FAK inhibitor 14, recombinant human epidermal growth factor (EGF), FITC (Fluorescein isothiocyanate)-conjugated Phalloidin (1:200 for IFA), and purified mouse IgG (1:200 for IFA) were purchased from Sigma-Aldrich Co. (St. Louis, MO, USA). Rhodamine-conjugated anti-mouse IgG antibody, rhodamine-conjugated anti-rabbit IgG antibody, FITC-conjugated anti-mouse IgG antibody, FITC-conjugated anti-rabbit IgG antibody (1:200 for IFA), and Horseradish peroxidase (HRP)-conjugated anti-mouse IgG antibody (1:10,000 for Western blot) were purchased from DiNonA (Seoul, Korea). HRP-conjugated goat anti-rabbit IgG antibody (1:10,000 for Western blot) was purchased from Chemicon (Temecula, CA, USA). Mouse monoclonal anti-human epidermal growth factor receptor (EGFR) antibody (1:150 for IFA), rabbit polyclonal anti-human EGFR antibody (1:150 for IFA), and rabbit polyclonal or mouse monoclonal anti-EEA1 antibody (1:150 for IFA) were purchased from Abcam (Cambridge, UK). Antibodies against HRAS, SHP2, PKA, Ezrin, WAVE2, Arp2, ROCK2, Grb2, Shc1, Rac1, RhoA, EGFR, β-actin, and HRP-conjugated donkey anti-goat IgG antibody (1:10,000 for Western blot) were obtained from Santa Cruz Biotechnology, Inc. (Santa Cruz, CA, USA). Antibodies against phospho-FAK (Tyr397), FAK, phospho-EGFR (Tyr1068), PTPN12, c-Src, HER2, and Gab1 were purchased from Cell Signaling Technology (Danvers, MA, USA).

### 4.2. Cell Culture

Human breast adenocarcinoma cell line MCF-7 was obtained from American Type Culture Collection (ATCC). Triple-negative breast carcinoma cell line MDA-MB-231 was kindly provided by Dr. Hyung Geun Song (DiNonA Inc., Seoul, Korea). MCF-7 cells were cultured in Dulbecco’s modified Eagle’s medium (DMEM) containing 10% (*v/v*) fetal bovine serum (FBS), 100 unit/mL penicillin, and 100 μg/mL streptomycin (Gibco-BRL, Grand Island, NY, USA). MDA-MB-231 cells were cultured in Roswell Park memorial Institute (RPMI) 1640 (10% FBS, 100 unit/mL penicillin, 100 μg/mL streptomycin, and 25 mM HEPES). All cells were maintained at 37 °C in a humidified 5% CO_2_ incubator.

### 4.3. Synthesis of Polypeptides

CD99 agonist polypeptide CD99CRIII3 was synthesized using an automatic peptide synthesizer (PeptrEx-R48, Peptron, Daejeon, Korea) according to the 9-fluorenylmethoxycarbonyl (Fmoc) solid-phase method. The synthesized polypeptides were purified and analyzed using reverse-phase high-performance liquid chromatography (Prominence LC-20AB, Shimadzu, Japan) equipped with a C18 analytical RP column (Capcell Pak column, Shiseido Co., Ltd., Japan). The mass was analyzed using a mass spectrometer (HP1100 Series LC/MSD, Hewlett-Packard, Roseville, CA, USA). The analytical results are described in Figure S3.

### 4.4. Plasmids and RNA Interference

The coding sequences of human WAVE2 and Arp2 were obtained by PCR with respective pairs of primers. Sense primer 5′-GGGGTACCGCCACCATGCCGTTAGTAACGAGGAAC-3′ and antisense primer 5′-GCTCTAGAGAGTTAATCGGACCAGTCGTC-3′ for the cDNA of WAVE2, sense primer 5′-GGGGTACCGCCACCATGGACAGCCAGGGCAGG-3′ and antisense primer 5′-GCTCTAGATTATCGAACAGTCACACCAAG-3′ for the cDNA of Arp2. Full length human WAVE2 and Arp2 cDNAs were subcloned into KpnI and XbaI sites of the pcDNA3 vector. The sequences of the constructs were confirmed by DNA sequencing. The expression vectors pEXV/constitutively active Rac1, pEXV/dominant negative Rac1, pEXV/constitutively active RhoA, pEXV/dominant negative RhoA, and pcDNA3.1/dominant negative c-Src were kindly donated by Dr. Hansoo Lee. The expression vector kinase-dead, non-phosphorylatable dominant negative FAK Y397F (pcDNA3/FAK Y397F) was kindly provided by Dr. Soo-Chul Park (Sookmyung Women’s University, Seoul, S. Korea). Constitutively active FAK plasmid (pCDM8/CD2-FAK) was kindly provided by Dr. Andrey V. Cybulsky (McGill University, Montreal, QC, Canada). The pCS2/ROCK2 vector was kindly provided by Dr. Anming Meng (Tsinghua University, Beijing, China). The pCB6/rsr-G-Tag/Ezrin vector was kindly provided by Dr. Janet Allopenna (Stony Brook Medicine, NY, USA). For gene knockdown experiments, the small interfering RNAs (siRNAs) against FAK, Shc1, c-Src, Arp2, Ezrin, PKA-α, SHP2, HRAS, PTPN12, and shRNA targeting PTPN12 were purchased from Santa Cruz Biotechnology, Inc. (Santa Cruz, CA, USA). Plasmids or siRNA duplexes were transfected into cells using Lipofectamine LTX/PLUS or RNAiMAX (Invitrogen Life Technologies, Grand Island, NY, USA). The PTPN12 knockdown MDA-MB-231 resistant clone was established by selecting with 0.4 mg/mL puromycin. After transfection, knockdown of each of the target molecules or expression of dominant negative or constitutively active DNA was confirmed by Western blotting.

### 4.5. In Situ Proximity Ligation Assay (PLA)

The in situ PLA analysis was performed using Duolink^®^ in situ reagents (O-LINK^®^ Bioscience, Uppsala, Sweden) according to the manufacturer’s instructions. Cells were transfected with plasmids or siRNAs and then seeded on glass coverslips in 24-well cell culture plates (1 × 10^5^ cells/well). After 24 h growth under standard conditions, cells were treated with EGF (25 ng/mL) in the presence or absence of peptides and each reagent for 15 min in a CO_2_ incubator at 37 °C, and then washed twice with 1X PBS. Cells were fixed with 2% formaldehyde in PBS for 10 min at room temperature (RT), and subsequently washed twice with 1X PBS, permeabilized with 0.1% Triton X-100 in PBS, and then washed twice with wash buffer A. Cells were incubated with blocking solution at 37 °C for 30 min, and then washed twice with wash buffer A. Cells were stained with specific antibodies (1:100 for in situ PLA) as indicated. Protein–protein interactions were analyzed using a confocal laser scanning microscope Olympus FluoView FV1000 (Olympus, Tokyo, Japan). PLA signals in cell populations (*n* = 4) were quantified by NIS-Elements analysis, and four or two independent experiments were performed. The average number of rolling-circle products (RCPs) per cell ± standard error is shown.

### 4.6. Dimerization Assay

BS^3^ [bis(sulfosuccinimidyl) suberate] was obtained from Thermo Scientific (Waltham, MA, USA) and used according to the manufacturer’s instruction and reference [59]. Cells were serum-starved in DMEM or RPMI containing 0.1% bovine serum albumin (BSA) and incubated in serum-free medium supplemented with human EGF in the presence or absence of CD99CRIII3 for 1 h at 4 °C (incubation on ice during ligand stimulation allows for ligand-induced receptor dimerization but inhibits receptor endocytosis). Cells were washed three times with ice-cold 1X Ca^2+^-, Mg^2+^-free PBS, then incubated with BS^3^ (2 mM) at 4 °C for 30 min and an additional 20 min at RT, followed by quenching with 1M of Tris (pH 7.5). Cells were lysed with 1% NP40 buffer containing 10 mM of β-mercaptoethanol. Cell lysates were subjected to SDS-PAGE and analyzed by Western blotting.

### 4.7. Active GTPase Detection

Active GTPase assay was performed using an active Rac1 or RhoA detection kit (Cell Signaling Technology, Danvers, MA, USA) according to the manufacturer’s instructions. Cells were rinsed with 1× ice-cold PBS, then lysed with 1× lysis/binding/wash buffer plus 1 mM PMSF. Whole cell lysate was harvested and incubated on ice for 5 min, and then centrifuged at 16,000× *g* at 4 °C for 15 min. The clear supernatant was transferred to a new tube and subjected to active GTPase assay. Glutathione resin slurry (50%, 100 μL) was added to a spin cup with a collection tube and the tube was centrifuged at 6000× *g* for 1 min. After washing the resin with 400 μL of 1× lysis/binding/wash buffer, 20 μg of GST-PAK1-p21 binding domain (PBD) (for GTP-bound Rac1) or GST-Rhotekin-Rho binding domain (RBD) (for GTP-bound RhoA) was added to the spin cup containing glutathione resin. The cell lysate was immediately transferred to the spin cup and vortexed. The reaction mixture was incubated at 4 °C for 1 h with gentle rocking. The active GTPase-bound GST resin was washed with 1X lysis/binding/wash buffer containing 1 mM PMSF. GTP-bound Rac1 or RhoA was eluted with 2X SDS reducing buffer containing 200 mM 1,4-dithiothreitol (DTT), followed by Western blotting with mouse anti-Rac1 mAb or rabbit anti-RhoA pAb.

### 4.8. Western Blot Analysis and Immunoprecipitation

Western blotting and immunoprecipitation were carried out as described previously [24]. Serum-starved breast carcinoma cells were treated with the appropriate reagents for 15 min at 37 °C, 5% CO_2_. Cells were harvested and lysed with 1% NP40 lysis buffer (1% Nonidet P40, 150 mM NaCl, 50 mM Tris-HCl (pH 8.0), 5 mM EDTA) containing 10 mM phenylmethylsulfonyl fluoride, 1 μg/mL pepstatin A, 10 μg/mL leupeptin, 1 μg/mL aprotinin, and 1 mM sodium orthovanadate. Cell extracts were subjected to SDS-PAGE and subsequently transferred to PVDF membranes. Immunoblotting was carried out with the indicated antibodies (1:1,000 for Western blot) to detect the target proteins.

For immunoprecipitation, cells were treated for the indicated time with each set of reagents at 37 °C, 5% CO_2_. Cells were harvested and lysed with PRO-PREP^TM^ protein extraction solution (iNtRON Biotechnology, Inc., Seongnam, Korea). After centrifugation at 13,000 rpm and 4 °C for 15 min, supernatants were collected, and then incubated overnight with the appropriate antibodies at 4 °C on a nutator. The immunoprecipitates were incubated with Protein A/G PLUS-Agarose (Santa Cruz Biotechnology, Inc., Santa Cruz, CA, USA) beads for 3 h at 4 °C on a rotator and washed with PRO-PREP^TM^ solution. The precipitates were eluted with 1× sample buffer (50 mM Tris-HCl (pH 6.8), 100 mM DTT, 2% SDS, 0.1% bromophenol blue, and 10% glycerol). Western blot analysis was carried out with the indicated antibodies to detect the target proteins.

### 4.9. Immunofluorescence Assay (IFA)

The EGFR distribution, cytoskeletal organization, and localization of the related proteins were detected by immunofluorescence assay. To determine the changes in RTK distribution, cytoskeletal organization, and localization of the related proteins, cells were seeded on round-shaped glass coverslips as described above. The following day, cells were treated with the appropriate reagents, then washed with ice-cold PBS and fixed with 4% paraformaldehyde (PFA) in 1× PBS for 10 min at RT. Subsequently, cells were washed twice with PBS, permeabilized for 5 min with 0.1% Triton X-100 in PBS. After washing with PBS, the cells were incubated with appropriate primary and secondary antibodies to detect the target proteins. Alternatively, cells were stained with 0.2 μM of FITC-conjugated phalloidin to detect the fibrous actin filaments. The stained cells were mounted onto slides with an aqueous mounting medium. Fluorescence images were acquired using a confocal microscope Olympus FluoView FV1000 (Olympus, Tokyo, Japan).

### 4.10. Proliferation Assay

Cell proliferation was assessed by the Cell Counting Kit-8 (CCK-8) assay or crystal violet staining method. Wild-type or CA-FAK-transfected cells were seeded at a density of 5 × 10^3^ cells per well in 96-well culture plates. After 24 h incubation, cells were treated with EGF (25 ng/mL) in the presence or absence of CD99CRIII3 (40 μM) or FAK inhibitor (25 μM) in 100 μL of serum-free medium (SFM) for 24 h, 48 h, and 72 h. At the indicated time points, cell proliferation was assessed using Cell Counting Kit-8 (CCK8, Dojindo Molecular Technologies, Inc., Rockville, MD, USA)-based assay or crystal violet staining method. CCK-8 colorimetric reactions were assessed by measuring the absorbance at 450 nm using a microplate reader (Versa Max, NY, USA). The images of crystal violet-stained cells were captured with a Nikon Eclipse TE2000-U (Nikon Instruments Inc., Melville, NY, USA).

### 4.11. Tumor Xenograft and Immunohistochemistry (IHC)

All animal experiments were performed in accordance with the Institutional Guidelines of the Animal Care and Use Committees (IACUC) of Kangwon National University. This research has been approved by IACUC of Kangwon National University on 26 October 2016 (KW-161020-1). MDA-MB-231 cells were cultured in complete Roswell Park Memorial Institute (RPMI) 1640 (10% FBS, 25 mM HEPES, 100 units/mL penicillin, and 100 μg/mL streptomycin) medium. Wild-type or PTPN12 shRNA-transfected MDA-MB-231 cells (5 × 10^6^ cells in 50 µL SFM/mouse) were mixed 1:1 (*v/v*) with Matrigel and subcutaneously injected into the right flanks of 6-week-old female BALB/c nude mice. One week after tumor cell inoculation, mice were divided into three groups of five mice each when the tumor size exceeded 5 mm in diameter (10 mg/kg or 20 mg/kg of body weight of CD99CRIII3 and PBS control). CD99CRIII3 was intraperitoneally administered to mice every day for 14 days. The tumor size was measured using a caliper every other day for 15 days. After 21 days of tumor cell inoculation, mice were sacrificed and the tumor masses were removed and weighed. After measuring the volume and weight, four tumor masses from each treatment group were paraffinized, sectioned with a microtome and stained with hematoxylin and eosin (H&E) following internal procedures. The effect of CD99CRIII3 on EGFR dimerization in tumor xenograft was assessed by in situ PLA assay according to the manufacturer’s instructions. Tumor sections were stained with anti-human EGFR and anti-phospho-EGFR (Tyr1068) antibodies. The z-stacks were generated from images taken at 0.2–0.4 μm intervals. Z-stack images were collected from three randomly selected fields per tumor. EGFR–EGFR interaction was quantified and analyzed by mean red intensity of automated measurements. Six tumors, one of each treatment, were frozen using liquid nitrogen, subjected to cryosectioning and stained with primary antibodies specific for EGFR, PTPN12, and ki67, followed by incubation with respective fluorescent secondary antibodies. Fluorescence images were analyzed using a confocal laser scanning microscope Olympus FluoView FV1000 and H&E staining images were captured using an Olympus BX50 microscope (Olympus, Tokyo, Japan).

### 4.12. Statistical Analysis

Values are given as mean ± standard deviation (SD). Statistical significance was determined by the Student’s *t*-test using the statistical analysis software GraphPad Prism (version 8.0; San Diego, CA, USA) and *p* < 0.05 was considered statistically significant. All experiments were conducted twice or more to minimize experimental error. The representative data are shown in the figures.

## 5. Conclusions

We demonstrated that CD99 activation regulates actin cytoskeleton dynamics through PTPN12/FAK/Rho/Rac axis, thereby suppressing EGFR activation and relevant tumor growth. We propose a schematic model illustrating the possible mechanism for the CD99 agonist ligand-induced suppression of EGFR activation (Figure 7H). CD99 acts as an upstream regulator of the PTPN12-mediated negative feedback loop for regulating ligand-induced dimerization or oligomerization of plasma membrane protein kinases, which are involved in tumor development and progression. Taken together, we propose that CD99 agonist ligands have potential as novel therapeutic drug candidates to suppress human breast carcinoma via inhibition of EGF-mediated EGFR signaling.

## Supplementary Materials

The following are available online at https://www.mdpi.com/2072-6694/12/10/2895/s1, Figure S1: c-Src/FAK plays an important role in actin cytoskeleton reorganization, resulting in EGFR dimerization and internalization, Figure S2: Binding of ligand to its receptor is essential to induce Rac1/RhoA-mediated EGFR dimerization, Figure S3: HPLC/MS chromatogram of CD99-derived agonistic peptide (CD99CRIII3), Figure S4: Functional validation of the equivalence of CD99 agonist ligands, Figure S5: PTPN12 plays a critical role in the dissociation of the EGFR-associated signaling complex induced by CD99CRIII3, Figure S6: CD99CRIII3 suppresses EGF-induced Rac1/RhoA GTPase signaling cascades via PTPN12, Figure S7: The physiological characteristics of shPTPN12-MDA-MB-231 cells, Figure S8: Uncropped western blot figures.

## References

[1] Freed D.M., Alvarado D., Lemmon M.A. (2015). Ligand regulation of a constitutively dimeric EGF receptor. Nat. Commun.

[2] Wang Q., Villeneuve G., Wang Z. (2005). Control of epidermal growth factor receptor endocytosis by receptor dimerization, rather than receptor kinase activation. EMBO Rep..

[3] Schlessinger J. (2002). Ligand-induced, receptor-mediated dimerization and activation of EGF receptor. Cell.

[4] Huang Y., Bharill S., Karandur D., Peterson S.M., Marita M., Shi X., Kaliszewski M.J., Smith A.W., Isacoff E.Y., Kuriyan J. (2016). Molecular basis for multimerization in the activation of the epidermal growth factor receptor. Elife.

[5] Purba E.R., Saita E.I., Maruyama I.N. (2017). Activation of the EGF Receptor by Ligand Binding and Oncogenic Mutations: The “Rotation Model”. Cells.

[6] Maruyama I.N. (2014). Mechanisms of activation of receptor tyrosine kinases: Monomers or dimers. Cells.

[7] Maruyama I.N. (2015). Activation of transmembrane cell-surface receptors via a common mechanism? The “rotation model”. Bioessays.

[8] Mansour M., Nievergall E., Gegenbauer K., Llerena C., Atapattu L., Halle M., Tremblay M.L., Janes P.W., Lackmann M. (2016). PTP-PEST controls EphA3 activation and ephrin-induced cytoskeletal remodelling. J. Cell Sci..

[9] Espejo R., Jeng Y., Paulucci-Holthauzen A., Rengifo-Cam W., Honkus K., Anastasiadis P.Z., Sastry S.K. (2014). PTP-PEST targets a novel tyrosine site in p120 catenin to control epithelial cell motility and Rho GTPase activity. J. Cell Sci..

[10] Noren N.K., Liu B.P., Burridge K., Kreft B. (2000). p120 catenin regulates the actin cytoskeleton via Rho family GTPases. J. Cell Biol..

[11] Anastasiadis P.Z., Moon S.Y., Thoreson M.A., Mariner D.J., Crawford H.C., Zheng Y., Reynolds A.B. (2000). Inhibition of RhoA by p120 catenin. Nat. Cell Biol..

[12] Grosheva I., Shtutman M., Elbaum M., Bershadsky A.D. (2001). p120 catenin affects cell motility via modulation of activity of Rho-family GTPases: A link between cell-cell contact formation and regulation of cell locomotion. J. Cell Sci..

[13] Sun T., Aceto N., Meerbrey K.L., Kessler J.D., Zhou C., Migliaccio I., Nguyen D.X., Pavlova N.N., Botero M., Huang J. (2011). Activation of multiple proto-oncogenic tyrosine kinases in breast cancer via loss of the PTPN12 phosphatase. Cell.

[14] Lee C., Rhee I. (2019). Important roles of protein tyrosine phosphatase PTPN12 in tumor progression. Pharm. Res..

[15] Nair A., Chung H.C., Sun T., Tyagi S., Dobrolecki L.E., Dominguez-Vidana R., Kurley S.J., Orellana M., Renwick A., Henke D.M. (2018). Combinatorial inhibition of PTPN12-regulated receptors leads to a broadly effective therapeutic strategy in triple-negative breast cancer. Nat. Med..

[16] Li J., Davidson D., Martins Souza C., Zhong M.C., Wu N., Park M., Muller W.J., Veillette A. (2015). Loss of PTPN12 Stimulates Progression of ErbB2-Dependent Breast Cancer by Enhancing Cell Survival, Migration, and Epithelial-to-Mesenchymal Transition. Mol. Cell Biol..

[17] Luo R.Z., Cai P.Q., Li M., Fu J., Zhang Z.Y., Chen J.W., Cao Y., Yun J.P., Xie D., Cai M.Y. (2014). Decreased expression of PTPN12 correlates with tumor recurrence and poor survival of patients with hepatocellular carcinoma. PLoS ONE.

[18] Lee K.J., Yoo Y.H., Kim M.S., Yadav B.K., Kim Y., Lim D., Hwangbo C., Moon K.W., Kim D., Jeoung D. (2015). CD99 inhibits CD98-mediated beta1 integrin signaling through SHP2-mediated FAK dephosphorylation. Exp. Cell Res..

[19] Lee K.J., Lee S.H., Yadav B.K., Ju H.M., Kim M.S., Park J.H., Jeoung D., Lee H., Hahn J.H. (2012). The activation of CD99 inhibits cell-extracellular matrix adhesion by suppressing beta(1) integrin affinity. BMB Rep..

[20] Guerzoni C., Fiori V., Terracciano M., Manara M.C., Moricoli D., Pasello M., Sciandra M., Nicoletti G., Gellini M., Dominici S. (2015). CD99 triggering in Ewing sarcoma delivers a lethal signal through p53 pathway reactivation and cooperates with doxorubicin. Clin. Cancer Res..

[21] Waclavicek M., Majdic O., Stulnig T., Berger M., Sunder-Plassmann R., Zlabinger G.J., Baumruker T., Stockl J., Ebner C., Knapp W. (1998). CD99 engagement on human peripheral blood T cells results in TCR/CD3-dependent cellular activation and allows for Th1-restricted cytokine production. J. Immunol..

[22] Sohn H.W., Shin Y.K., Lee I.S., Bae Y.M., Suh Y.H., Kim M.K., Kim T.J., Jung K.C., Park W.S., Park C.S. (2001). CD99 regulates the transport of MHC class I molecules from the Golgi complex to the cell surface. J. Immunol..

[23] Sohn H.W., Choi E.Y., Kim S.H., Lee I.S., Chung D.H., Sung U.A., Hwang D.H., Cho S.S., Jun B.H., Jang J.J. (1998). Engagement of CD99 induces apoptosis through a calcineurin-independent pathway in Ewing’s sarcoma cells. Am. J. Pathol..

[24] Lee K.J., Kim Y., Yoo Y.H., Kim M.S., Lee S.H., Kim C.G., Park K., Jeoung D., Lee H., Ko I.Y. (2017). CD99-Derived Agonist Ligands Inhibit Fibronectin-Induced Activation of beta1 Integrin through the Protein Kinase A/SHP2/Extracellular Signal-Regulated Kinase/PTPN12/Focal Adhesion Kinase Signaling Pathway. Mol. Cell Biol..

[25] Zucchini C., Manara M.C., Pinca R.S., De Sanctis P., Guerzoni C., Sciandra M., Lollini P.L., Cenacchi G., Picci P., Valvassori L. (2014). CD99 suppresses osteosarcoma cell migration through inhibition of ROCK2 activity. Oncogene.

[26] Zhao T.T., Le Francois B.G., Goss G., Ding K., Bradbury P.A., Dimitroulakos J. (2010). Lovastatin inhibits EGFR dimerization and AKT activation in squamous cell carcinoma cells: Potential regulation by targeting rho proteins. Oncogene.

[27] Mattila P.K., Batista F.D., Treanor B. (2016). Dynamics of the actin cytoskeleton mediates receptor cross talk: An emerging concept in tuning receptor signaling. J. Cell Biol..

[28] Low-Nam S.T., Lidke K.A., Cutler P.J., Roovers R.C., van Bergen en Henegouwen P.M., Wilson B.S., Lidke D.S. (2011). ErbB1 dimerization is promoted by domain co-confinement and stabilized by ligand binding. Nat. Struct Mol. Biol..

[29] Ren X.D., Kiosses W.B., Sieg D.J., Otey C.A., Schlaepfer D.D., Schwartz M.A. (2000). Focal adhesion kinase suppresses Rho activity to promote focal adhesion turnover. J. Cell Sci..

[30] Zhai J., Lin H., Nie Z., Wu J., Canete-Soler R., Schlaepfer W.W., Schlaepfer D.D. (2003). Direct interaction of focal adhesion kinase with p190RhoGEF. J. Biol Chem..

[31] Westhoff M.A., Serrels B., Fincham V.J., Frame M.C., Carragher N.O. (2004). SRC-mediated phosphorylation of focal adhesion kinase couples actin and adhesion dynamics to survival signaling. Mol. Cell Biol..

[32] Suetsugu S., Takenawa T. (2003). Regulation of cortical actin networks in cell migration. Int. Rev. Cytol..

[33] Yamazaki D., Kurisu S., Takenawa T. (2005). Regulation of cancer cell motility through actin reorganization. Cancer Sci..

[34] Spiering D., Hodgson L. (2011). Dynamics of the Rho-family small GTPases in actin regulation and motility. Cell Adh. Migr..

[35] Bai C.Y., Ohsugi M., Abe Y., Yamamoto T. (2007). ZRP-1 controls Rho GTPase-mediated actin reorganization by localizing at cell-matrix and cell-cell adhesions. J. Cell Sci..

[36] Fraguas S., Barberan S., Cebria F. (2011). EGFR signaling regulates cell proliferation, differentiation and morphogenesis during planarian regeneration and homeostasis. Dev. Biol..

[37] Song Z., Fusco J., Zimmerman R., Fischbach S., Chen C., Ricks D.M., Prasadan K., Shiota C., Xiao X., Gittes G.K. (2016). Epidermal Growth Factor Receptor Signaling Regulates beta Cell Proliferation in Adult Mice. J. Biol. Chem..

[38] Pennock S., Wang Z. (2003). Stimulation of cell proliferation by endosomal epidermal growth factor receptor as revealed through two distinct phases of signaling. Mol. Cell Biol..

[39] Zheng Y., Zhang C., Croucher D.R., Soliman M.A., St-Denis N., Pasculescu A., Taylor L., Tate S.A., Hardy W.R., Colwill K. (2013). Temporal regulation of EGF signalling networks by the scaffold protein Shc1. Nature.

[40] Ayoub E., Hall A., Scott A.M., Chagnon M.J., Miquel G., Halle M., Noda M., Bikfalvi A., Tremblay M.L. (2013). Regulation of the Src kinase-associated phosphoprotein 55 homologue by the protein tyrosine phosphatase PTP-PEST in the control of cell motility. J. Biol. Chem..

[41] Trimble W.S., Grinstein S. (2015). Barriers to the free diffusion of proteins and lipids in the plasma membrane. J. Cell Biol..

[42] Jung S.R., Seo J.B., Shim D., Hille B., Koh D.S. (2012). Actin cytoskeleton controls movement of intracellular organelles in pancreatic duct epithelial cells. Cell Calcium.

[43] Manneville J.B., Etienne-Manneville S., Skehel P., Carter T., Ogden D., Ferenczi M. (2003). Interaction of the actin cytoskeleton with microtubules regulates secretory organelle movement near the plasma membrane in human endothelial cells. J. Cell Sci..

[44] Tang J., Gross D.J. (2003). Regulated EGF receptor binding to F-actin modulates receptor phosphorylation. Biochem. Biophys. Res. Commun..

[45] Den Hartigh J.C., van Bergen en Henegouwen P.M., Verkleij A.J., Boonstra J. (1992). The EGF receptor is an actin-binding protein. J. Cell Biol..

[46] Martinez-Munoz L., Rodriguez-Frade J.M., Barroso R., Sorzano C.O.S., Torreno-Pina J.A., Santiago C.A., Manzo C., Lucas P., Garcia-Cuesta E.M., Gutierrez E. (2018). Separating Actin-Dependent Chemokine Receptor Nanoclustering from Dimerization Indicates a Role for Clustering in CXCR4 Signaling and Function. Mol. Cell.

[47] Carragher N.O., Frame M.C. (2004). Focal adhesion and actin dynamics: A place where kinases and proteases meet to promote invasion. Trends Cell Biol..

[48] Wehrle-Haller B. (2012). Assembly and disassembly of cell matrix adhesions. Curr. Opin. Cell Biol..

[49] Li S.Y., Mruk D.D., Cheng C.Y. (2013). Focal adhesion kinase is a regulator of F-actin dynamics: New insights from studies in the testis. Spermatogenesis.

[50] Rae J.M., Scheys J.O., Clark K.M., Chadwick R.B., Kiefer M.C., Lippman M.E. (2004). EGFR and EGFRvIII expression in primary breast cancer and cell lines. Breast Cancer Res. Treat..

[51] Manara M.C., Terracciano M., Mancarella C., Sciandra M., Guerzoni C., Pasello M., Grilli A., Zini N., Picci P., Colombo M.P. (2016). CD99 triggering induces methuosis of Ewing sarcoma cells through IGF-1R/RAS/Rac1 signaling. Oncotarget.

[52] Stock J. (1996). Receptor signaling: Dimerization and beyond. Curr. Biol..

[53] Weiss A., Schlessinger J. (1998). Switching signals on or off by receptor dimerization. Cell.

[54] Rowland-Goldsmith M.A., Maruyama H., Kusama T., Ralli S., Korc M. (2001). Soluble type II transforming growth factor-beta (TGF-beta) receptor inhibits TGF-beta signaling in COLO-357 pancreatic cancer cells in vitro and attenuates tumor formation. Clin. Cancer Res..

[55] Farooqi A.A., Siddik Z.H. (2015). Platelet-derived growth factor (PDGF) signalling in cancer: Rapidly emerging signalling landscape. Cell Biochem. Funct..

[56] Helsten T., Elkin S., Arthur E., Tomson B.N., Carter J., Kurzrock R. (2016). The FGFR Landscape in Cancer: Analysis of 4,853 Tumors by Next-Generation Sequencing. Clin. Cancer Res..

[57] Zou L., Cao S., Kang N., Huebert R.C., Shah V.H. (2012). Fibronectin induces endothelial cell migration through beta1 integrin and Src-dependent phosphorylation of fibroblast growth factor receptor-1 at tyrosines 653/654 and 766. J. Biol Chem..

[58] Chen M., She H., Kim A., Woodley D.T., Li W. (2000). Nckbeta adapter regulates actin polymerization in NIH 3T3 fibroblasts in response to platelet-derived growth factor bb. Mol. Cell Biol..

[59] Turk H.F., Chapkin R.S. (2015). Analysis of epidermal growth factor receptor dimerization by BS(3) cross-linking. Methods Mol. Biol..

