# Supplementary material for: CD99–PTPN12 Axis Suppresses Actin Cytoskeleton-Mediated Dimerization of Epidermal Growth Factor Receptor
Source: Cancers (Basel). 2020 Oct 9;12(10):2895. doi: 10.3390/cancers12102895 (PMC7599698; doi:10.3390/cancers12102895)
Supplement: Supplementary file 1 [file cancers-12-02895-s001.pdf]

Supplementary Material: CD99–PTPN12 Axis Suppresses Actin Cytoskeleton-Mediated Dimerization of Epidermal Growth Factor Receptor

Kyoung-Jin Lee, Yuri Kim, Min Seo Kim, Hyun-Mi Ju, Boyoung Choi, Hansoo Lee, Dooil Jeoung, Ki-Won Moon, Dongmin Kang, Jiwon Choi, Jong In Yook and Jang-Hee Hanh

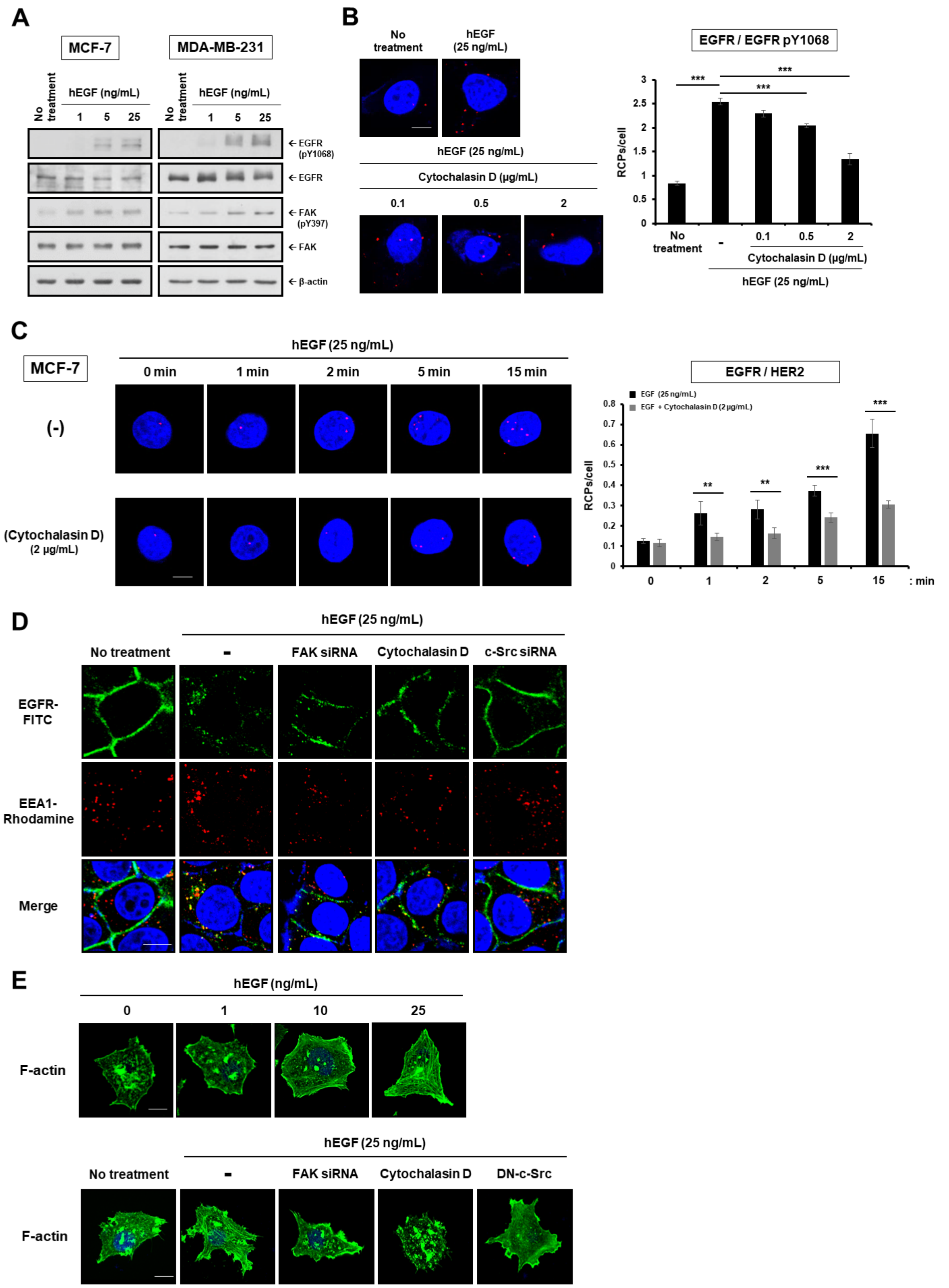

Figure S1. c-Src/FAK plays an important role in actin cytoskeleton reorganization, resulting in EGFR dimerization and internalization. (**A**) The expression and phosphorylation levels of proteins were analyzed by western blotting with the antibodies indicated. (**B**) To determine the appropriate concentration of cytochalasin D treatment, MCF-7 cells were incubated for 15 min at 37 °C with increasing concentrations of cytochalasin D (0.1–2 μg/mL) in the presence of EGF (25 ng/mL). The physical proximity of EGFR molecules was assessed by in situ proximity ligation assay (PLA). The average number of rolling-circle products (RCPs) per cell ± the standard error is shown. Asterisks represent statistically significant differences between treatments as follows: *** $p < 0.001$. (**C**) MCF-7

cells were treated with EGF (25 ng/mL) with or without 2 μg/mL of cytochalasin D. The dimerization levels of EGFR/HER2 at each time point were assessed by in situ PLA. ** $p < 0.01$; *** $p < 0.001$. (**D,E**) Dominant negative c-Src (DN-c-Src) or siRNA (targeting c-Src or FAK)-transfected MCF-7 cells were treated with EGF (25 ng/mL) for 15 min in the presence or absence of 2 μg/mL of cytochalasin D. EGFR endocytosis and actin cytoskeleton organization were determined by immunofluorescence assay (IFA). To confirm the intracellular localization of dimerized EGFR, cells were stained sequentially with mouse anti-EEA1 mAb and rabbit anti-human EGFR pAb, followed by FITC-conjugated anti-rabbit IgG and rhodamine-conjugated anti-mouse IgG. To detect the fibrous actin filament, cells were fixed with 4% paraformaldehyde and stained with 0.2 μM of FITC-Phalloidin. Original magnification of representative images, 600×. Scale bars = 10 μm.

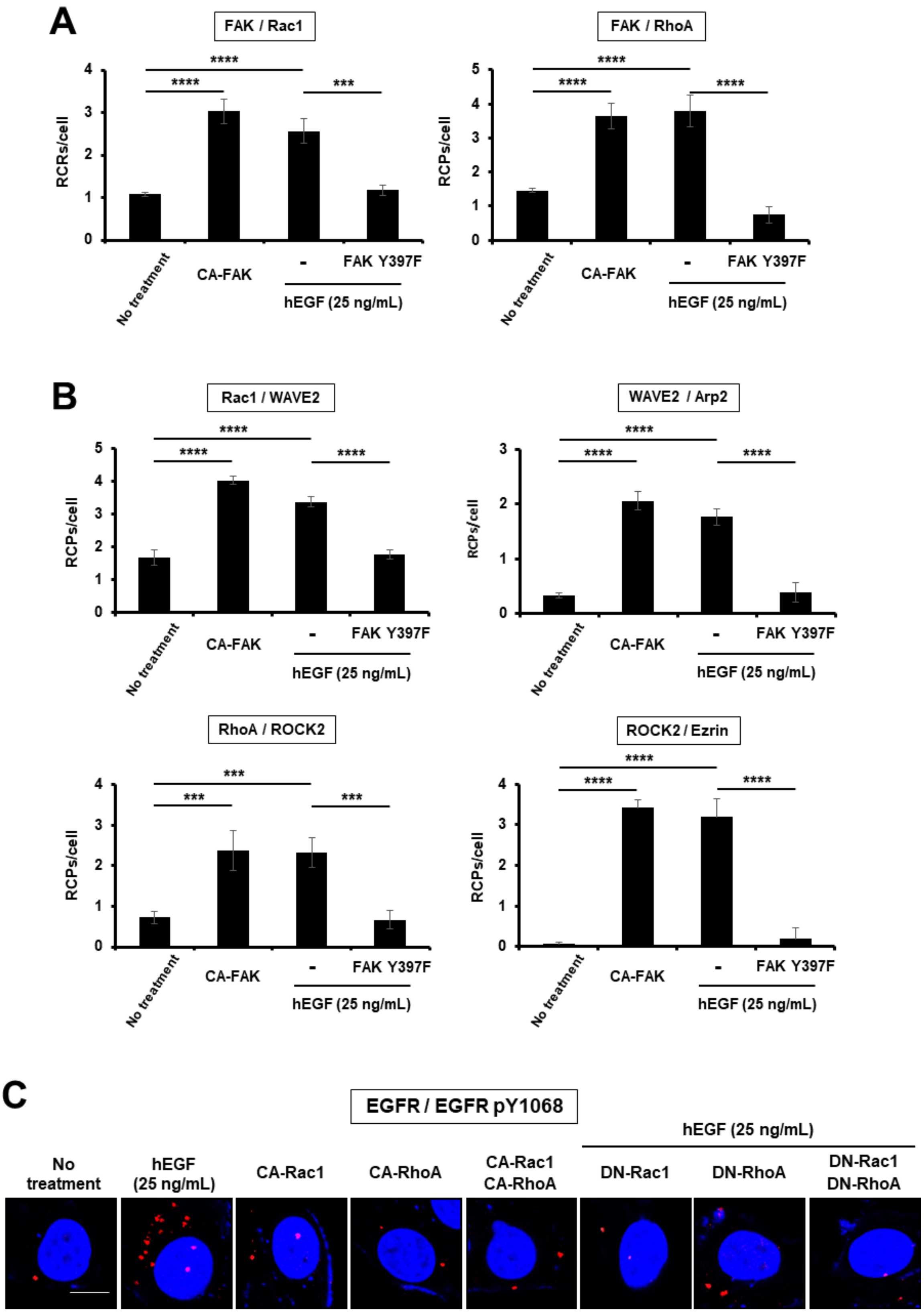

Figure S2. Binding of ligand to its receptor is essential to induce Rac1/RhoA-mediated EGFR dimerization. (**A**,**B**) MCF-7 cells were transfected with CA-FAK or FAK Y397F plasmids and subsequently incubated in the presence or absence of 25 ng/mL of EGF for 15 min at 37 °C, 5% CO_2. The interactions between the pairs of molecules indicated were assessed by *in situ* PLA. *** $p < 0.001$; **** $p < 0.0001$. (**C**) MCF-7 cells were transfected with expression plasmids (CA-Rac1, CA-RhoA, DN-

Rac1, DN-RhoA) and subsequently treated with 25 ng/mL of EGF for 15 min at 37 °C, 5% CO_2. EGFR dimerization was assessed by in situ PLA. Scale bars = 10 µm (600×).

HPLC analysis

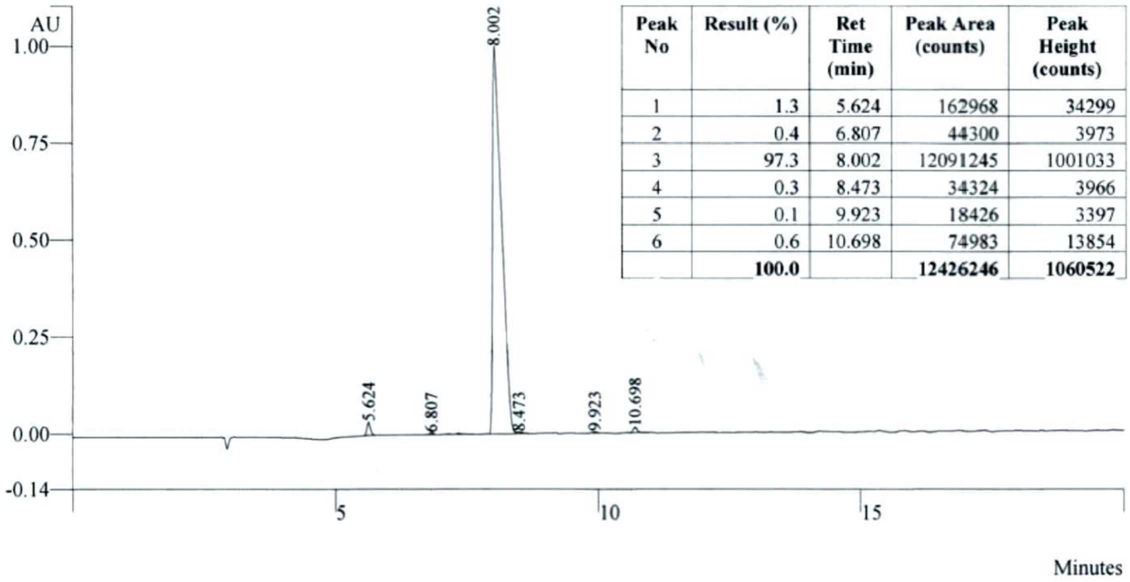

Peak No	Result (%)	Ret Time (min)	Peak Area (counts)	Peak Height (counts)
1	1.3	5.624	162968	34299
2	0.4	6.807	44300	3973
3	97.3	8.002	12091245	1001033
4	0.3	8.473	34324	3966
5	0.1	9.923	18426	3397
6	0.6	10.698	74983	13854
	**100.0**		**12426246**	**1060522**

MS analysis

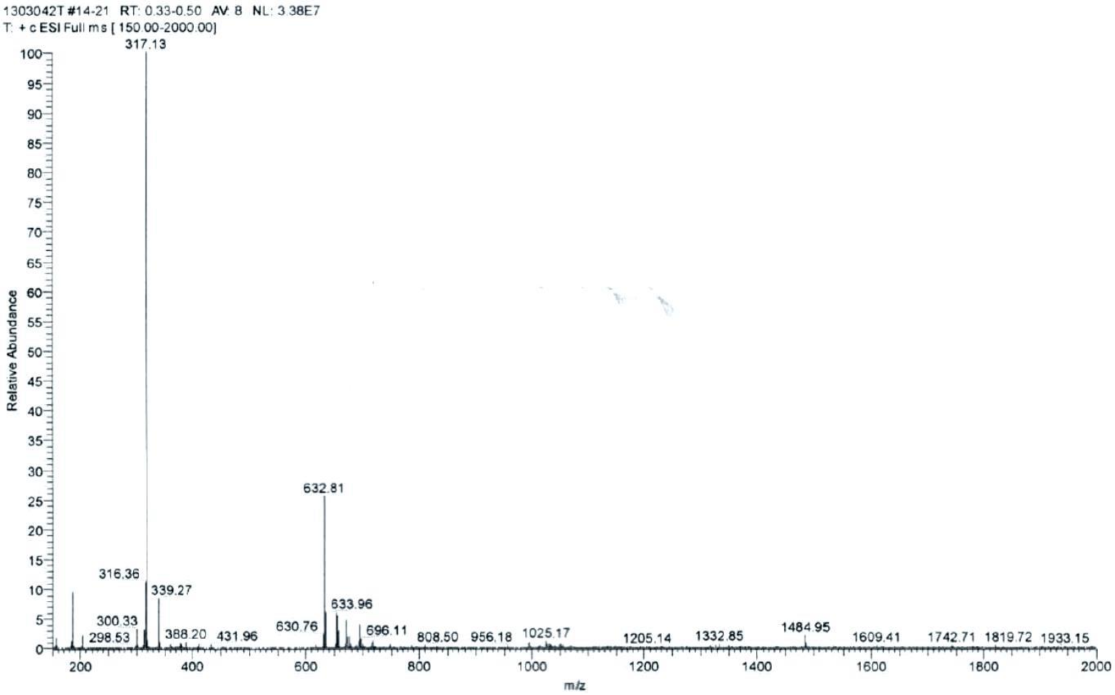

Figure S3. HPLC/MS chromatogram of CD99-derived agonistic peptide (CD99CRIII3). Detection of the synthetic tripeptide [leu(D)-ala(D)-asp(D)-NH2]. CD99CRIII3 tripeptide was synthesized using an automatic peptide synthesizer as described in Materials and Methods. It was purified and analyzed using reverse-phase high-performance liquid chromatography (HPLC/RP) equipped with a C18 analytical RP column and using a mass spectrometer (MS) to determine the purity and yield of the synthesized tripeptide.

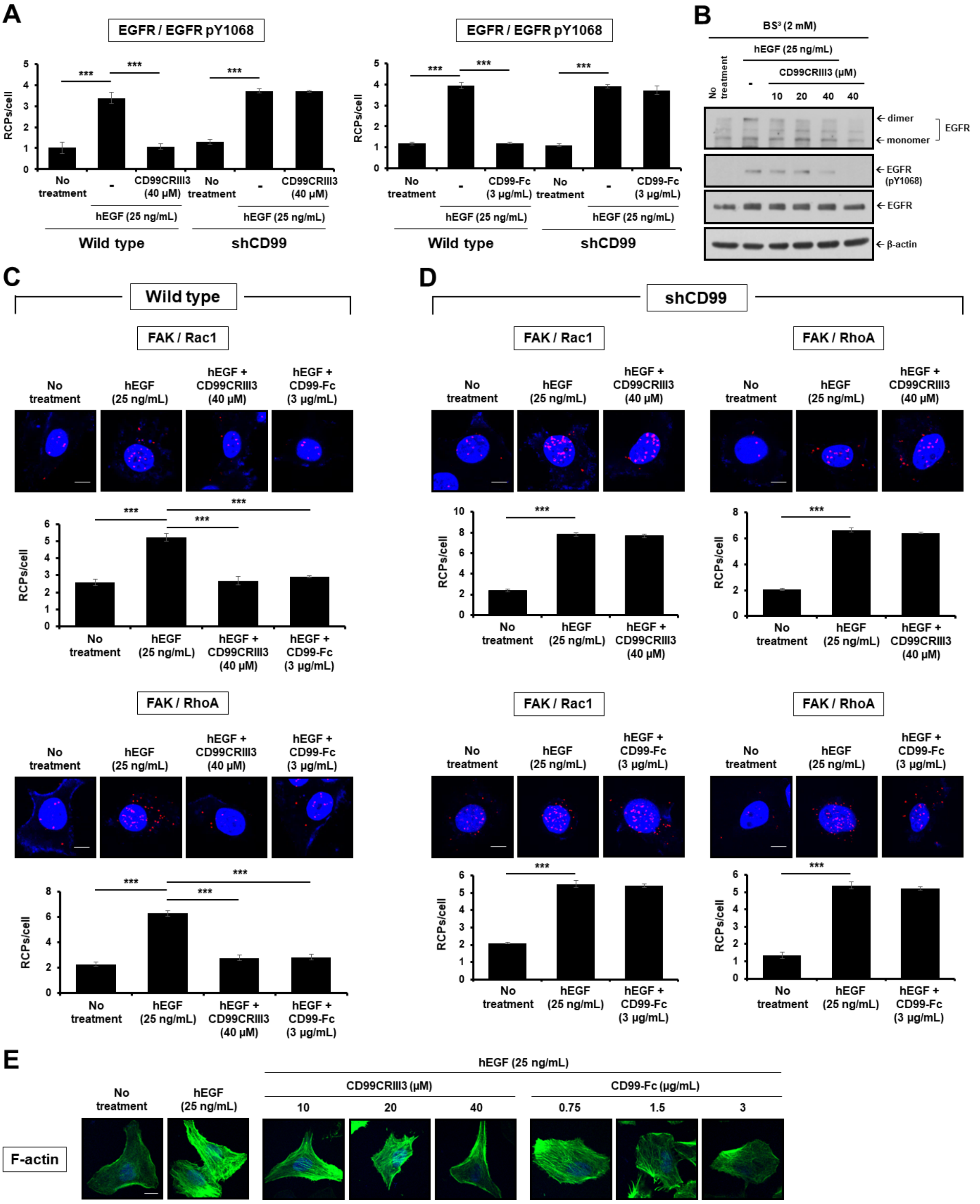

Figure S4. Functional validation of the equivalence of CD99 agonist ligands. (**A**,**C**,**D**) Wild-type and CD99 shRNA-transfected MCF-7 cells were treated with 25 ng/mL of EGF in the presence or absence of CD99CRIII3 or CD99-Fc for 15 min at 37 °C, 5% CO_2. The interactions between the pairs of molecules indicated were assessed by in situ PLA. *** $p < 0.001$. Scale bars = 10 μm (600×). (**B**) To determine EGFR dimerization, MCF-7 cells were treated with increasing concentrations of CD99CRIII3 in the presence or absence of EGF (25 ng/mL) for 1 h on ice, as described in the Materials and Methods. Cells were subjected to BS3 chemical-mediated crosslinking. To examine the phosphorylation level of EGFR at Y1068, cells were incubated in serum-free medium (SFM) with EGF and CD99CRIII3 for 15 min at 37 °C. Cell extracts were assessed via western blotting to determine the dimerization and phosphorylation levels of EGFR. (**E**) MDA-MB-231 cells were treated with increasing concentrations of CD99CRIII3 or CD99-Fc in the presence of EGF (25 ng/mL) for 15 min at

37 °C, 5% CO_2. Cells were stained with 0.2 μM of FITC-Phalloidin after fixation with 4% paraformaldehyde to examine the formation of fibrous actin cytoskeleton. Scale bars = 10 μm (600×).

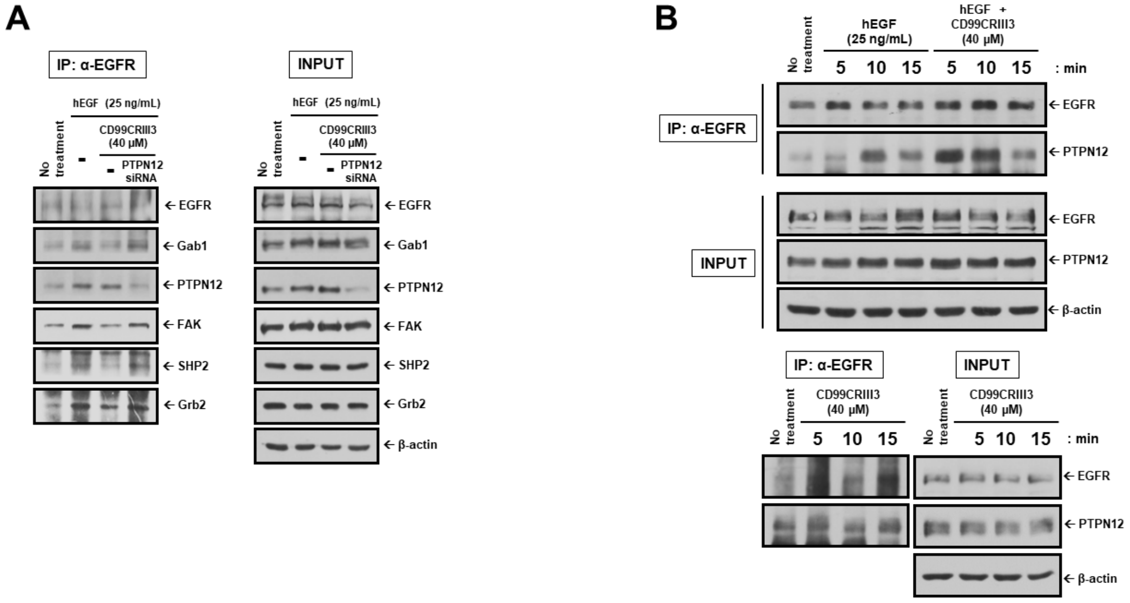

Figure S5. PTPN12 plays a critical role in the dissociation of the EGFR-associated signaling complex induced by CD99CRIII3. Cell lysates of wild-type or siRNA-transfected MCF-7 cells were immunoprecipitated with polyclonal anti-EGFR antibody. The immunoprecipitates were analyzed by performing western blot with the indicated antibodies. (**A**) MCF-7 cells were transfected with PTPN12 siRNA, followed by treatment with EGF (25 ng/mL) with or without CD99CRIII3 (40 μM) for 15 min. (**B**) MCF-7 cells were treated with EGF and/or CD99CRIII3 in a time-dependent manner.

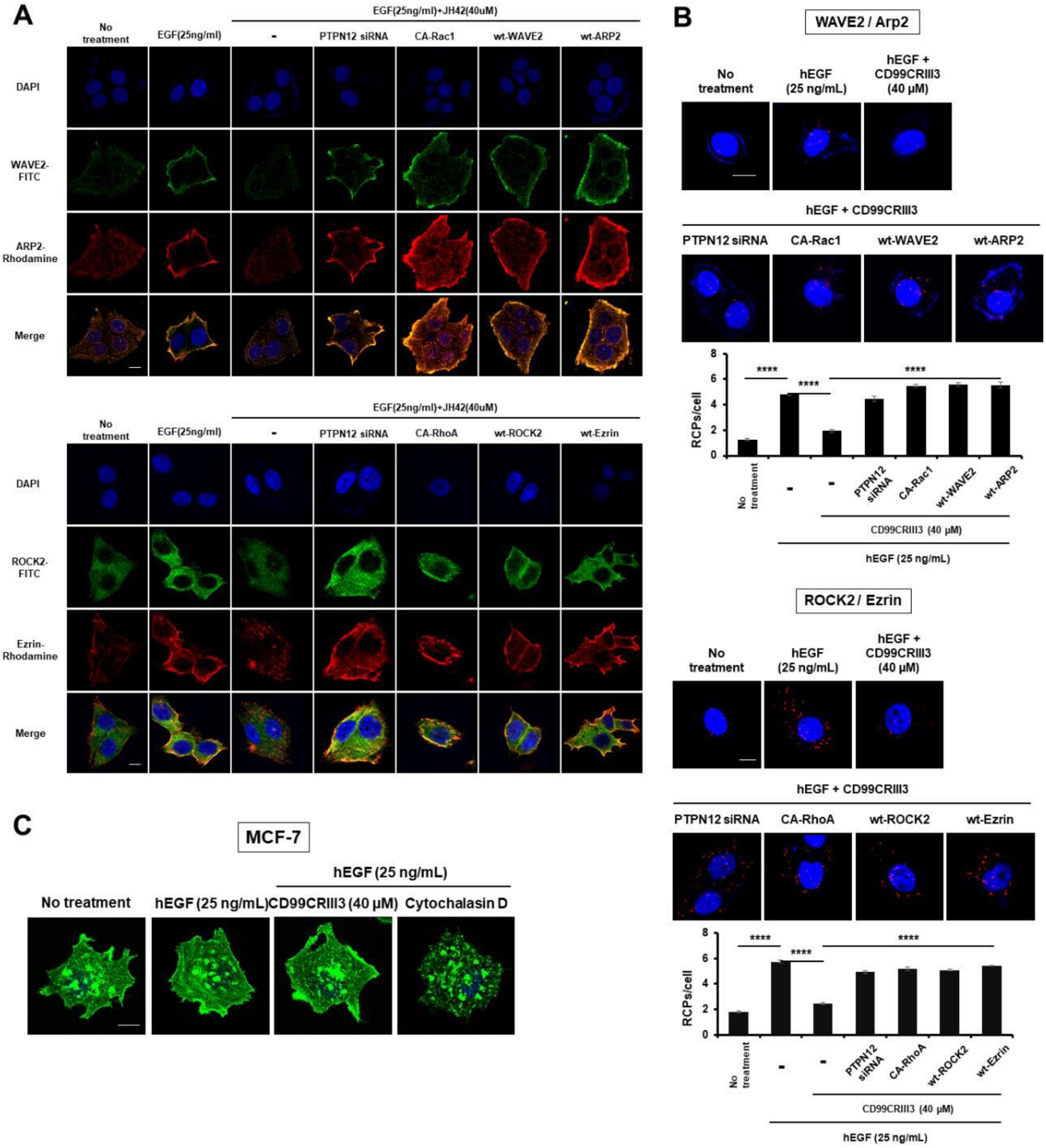

Figure S6. CD99CRIII3 suppresses EGF-induced Rac1/RhoA GTPase signaling cascades via PTPN12. (**A**,**B**) MCF-7 cells were transiently transfected with PTPN12 siRNA or expression plasmids for CA-Rac1, CA-RhoA, full-length of WAVE2, Arp2, ROCK2, and Ezrin. After 24 h of transfection, cells were seeded onto round-shaped coverslips and cultured for 24 h at 37 °C in 5% CO_2. Cells were incubated in SFM supplemented with 25 ng/ml of EGF in the presence or absence of CD99CRIII3 (40 μM) for 15 min. (**A**) Cells were sequentially stained with primary antibodies (goat anti-WAVE2 pAb, rabbit anti-Arp2 pAb, mouse anti-ROCK2 mAb, and rabbit anti-Ezrin pAb) and secondary antibodies (FITC-conjugated anti-goat IgG, rhodamine-conjugated anti-rabbit IgG, and FITC-conjugated anti-mouse IgG). Colocalization of each of the molecules was analyzed by confocal microscopy. (**B**) The interactions between the pairs of molecules indicated were assessed by *in situ* PLA. **** $p < 0.0001$. The representative images are shown. (**C**) MCF-7 cells were treated with EGF with or without CD99CRIII3 or cytochalasin D (2 μg/mL) for 15 min at 37 °C in 5% CO_2. Organization of actin cytoskeleton was determined by IFA as described above. Scale bars = 10 μm (600×).

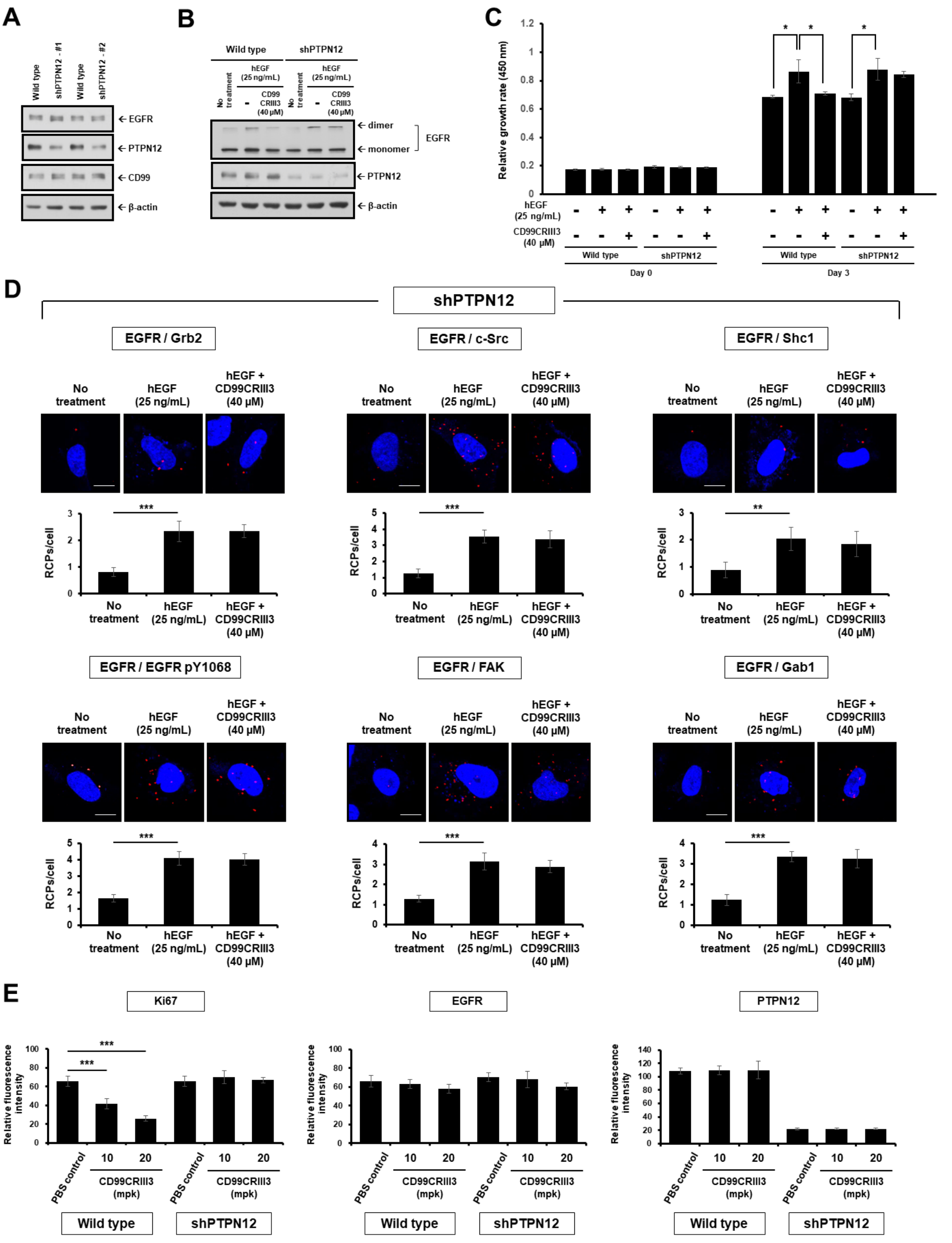

Figure S7. The physiological characteristics of shPTPN12-MDA-MB-231 cells. (**A**,**B**) MDA-MB-231 cells were transfected with shRNA plasmid specific for PTPN12. To determine EGFR dimerization, cells were treated with EGF with or without CD99CRIII3 for 1 h on ice, as described above. Cells were subjected to BS3 chemical-mediated crosslinking. To examine the expression levels of EGFR, PTPN12, and CD99, cells were incubated in SFM supplemented with CD99CRIII3 and/or EGF for 15 min at 37 °C. Cell lysates were assessed via western blotting to determine the dimerization and expression of EGFR and to verify the expression level of PTPN12 and CD99. (**C**) Cells were suspended in complete RPMI1640 medium with or without EGF or CD99CRIII3, seeded in a 96-well plate in triplicates, and allowed to grow for 3 days at 37 °C in 5% CO_2. Changes in growth rate were monitored for 3 days using CCK-8 assay kit. * $p < 0.05$ (**D**) shPTPN12-MDA-MB-231 cells were treated as indicated above.

The interactions between the pairs of molecules indicated were assessed by *in situ* PLA. ** $p < 0.01$; *** $p < 0.001$. Scale bars = 10 μm (600×). (**E**) Fluorescence signals in histological sections of tumor tissues were detected in nine randomly selected fields and the intensities were quantified by NIS-Elements analysis. *** $p < 0.001$.

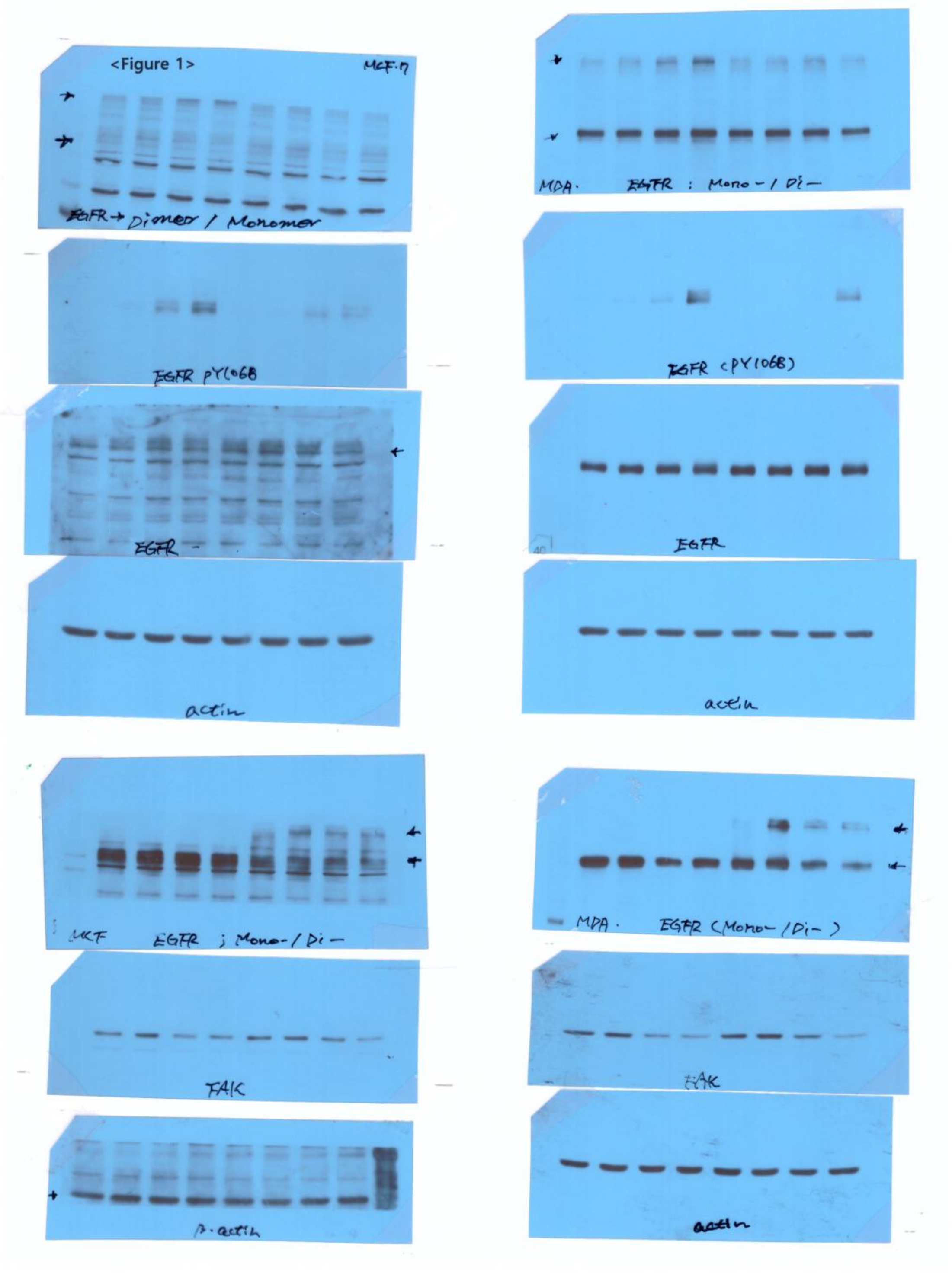

<Figure 2>

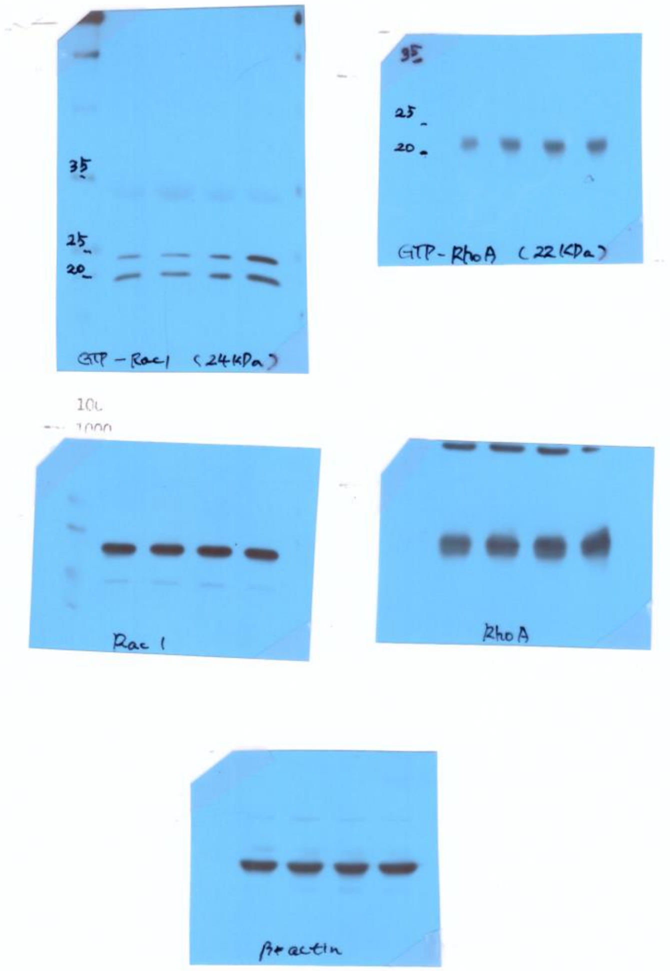
35
25
20
GTP - Rac1 (24kDa)
35
25
20
GTP - RhoA (22kDa)
Rac1
RhoA
β-actin

<Figure 2>

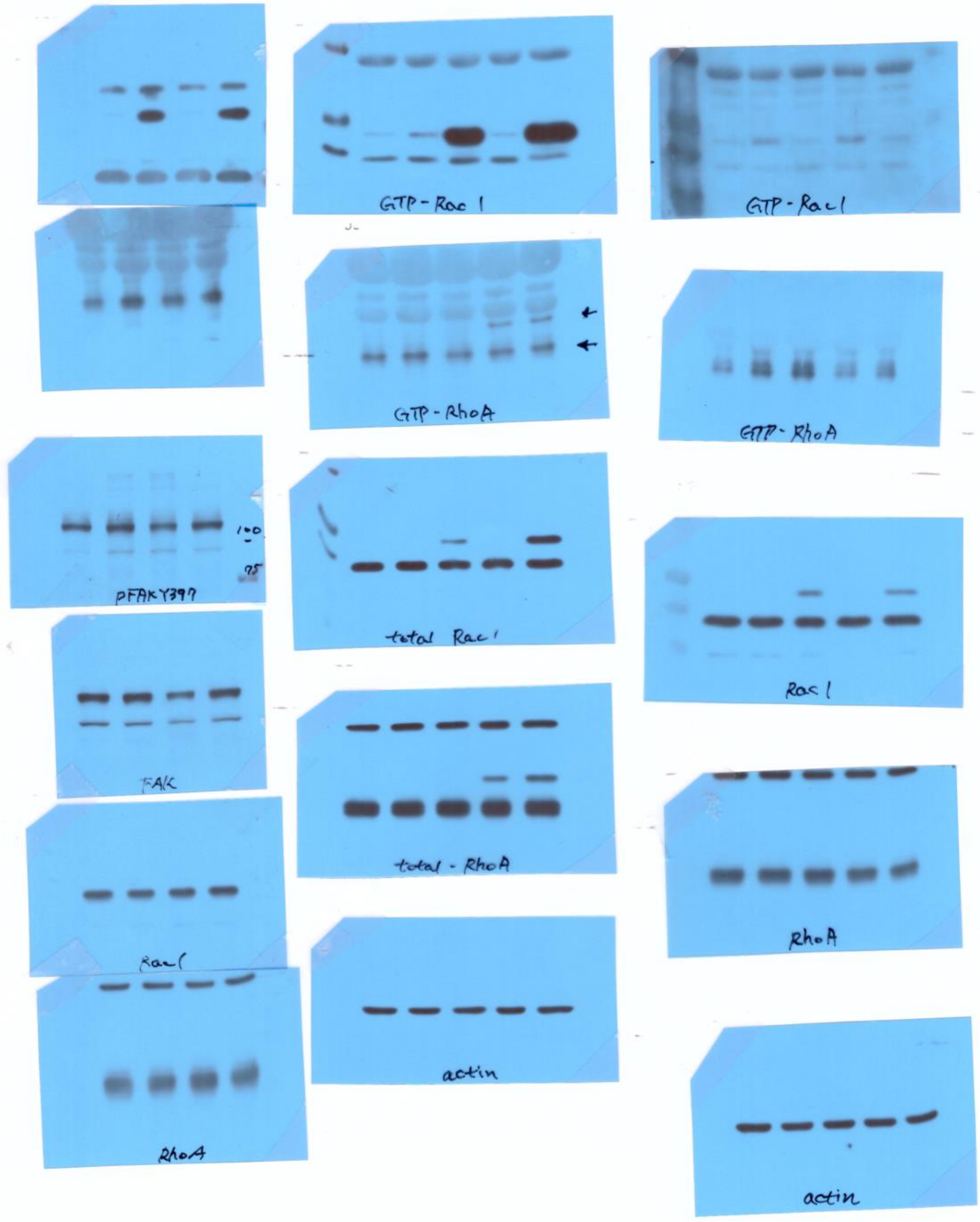
GTP-Rac1
GTP-Rac1
GTP-RhoA
GTP-RhoA
100
75
pFAKY397
total Rac1
Rac1
FAK
total-RhoA
RhoA
Rac1
RhoA
actin
actin

<Figure 3>

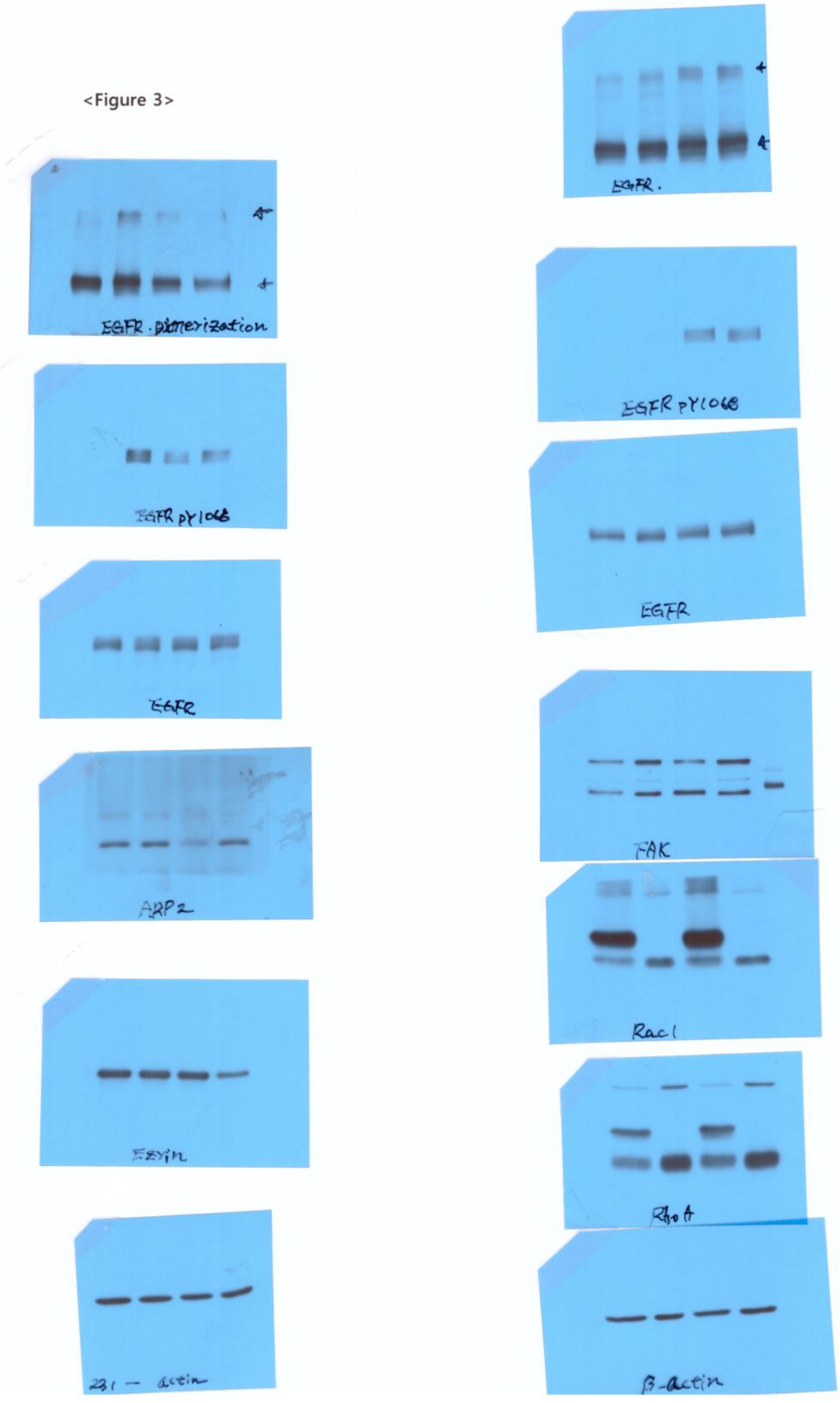
EGFR dimerization
EGFR pY1068
EGFR
ARP2
Ezrin
231 – actin
EGFR.
EGFR pY1068
EGFR
FAK
Rac1
RhoA
β-actin

<Figure 4>

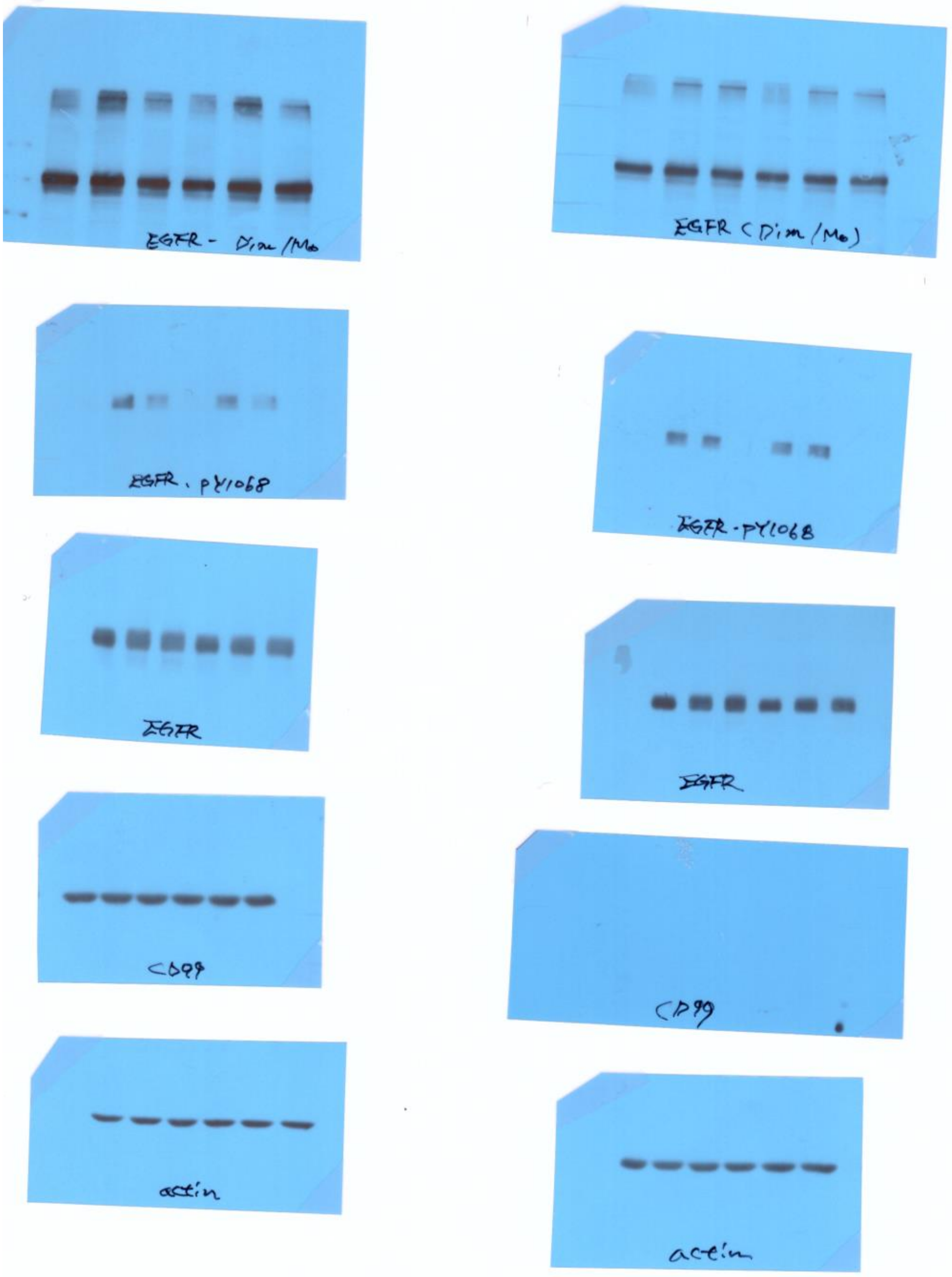
EGFR - Dim/Mo
EGFR (Dim/Mo)
EGFR. pY1068
EGFR-pY1068
EGFR
EGFR
CD99
CD99
actin
actin

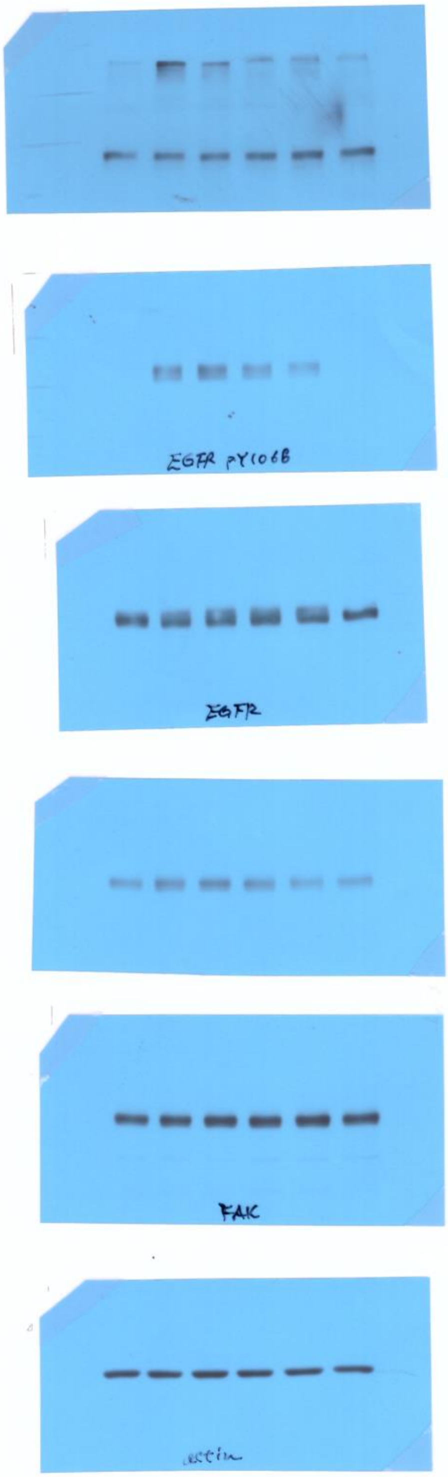
EGFR pY1068
EGFR
FAK
actin

<Figure 4>

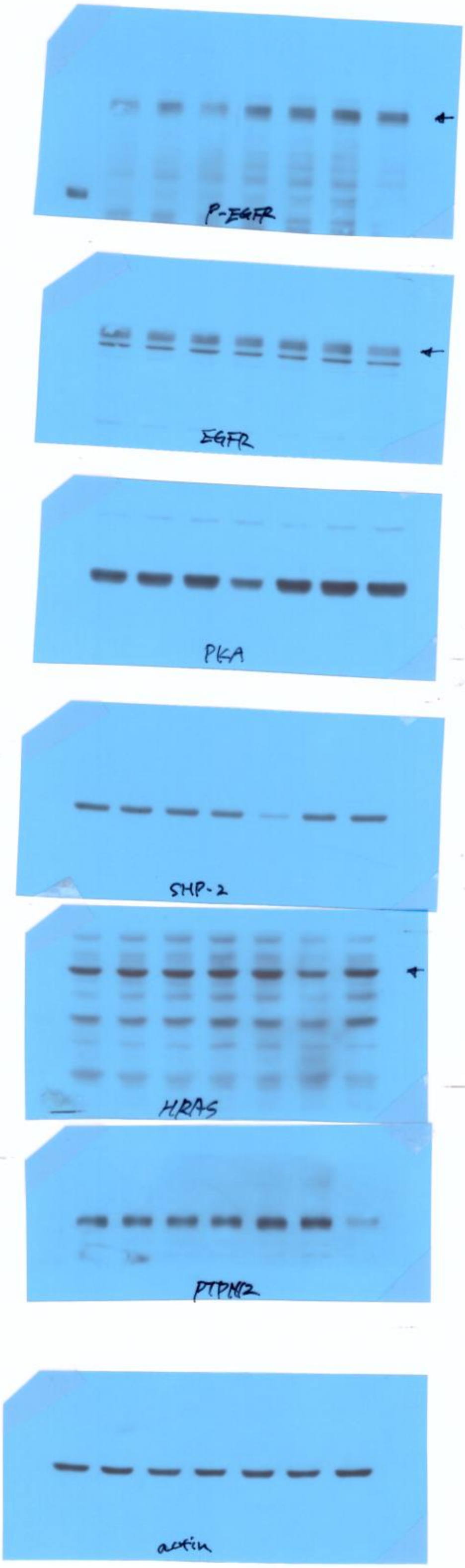

<Figure 5>

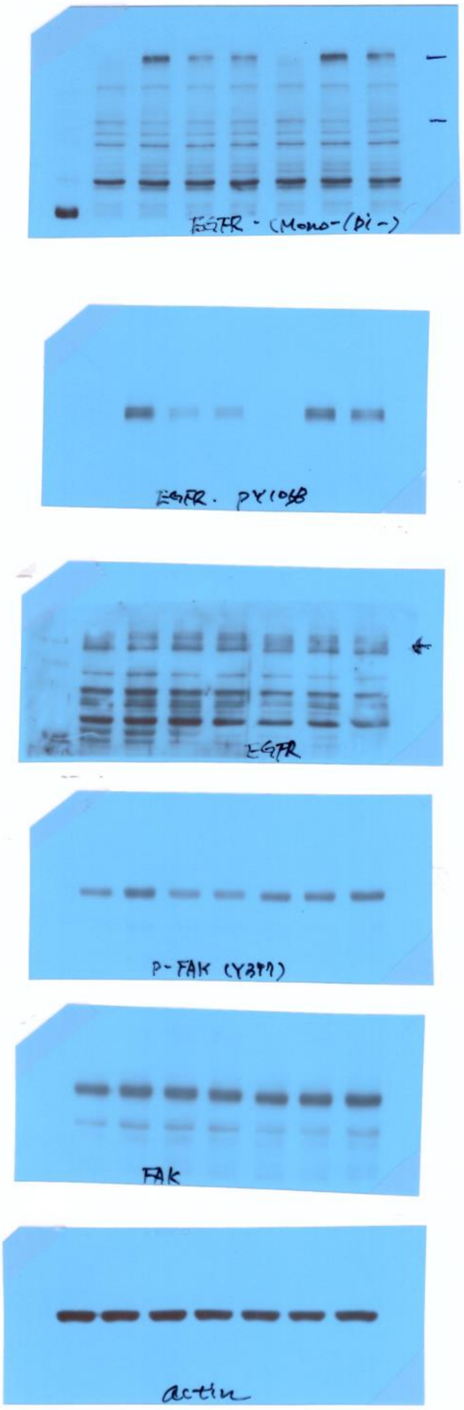

<Figure 6>

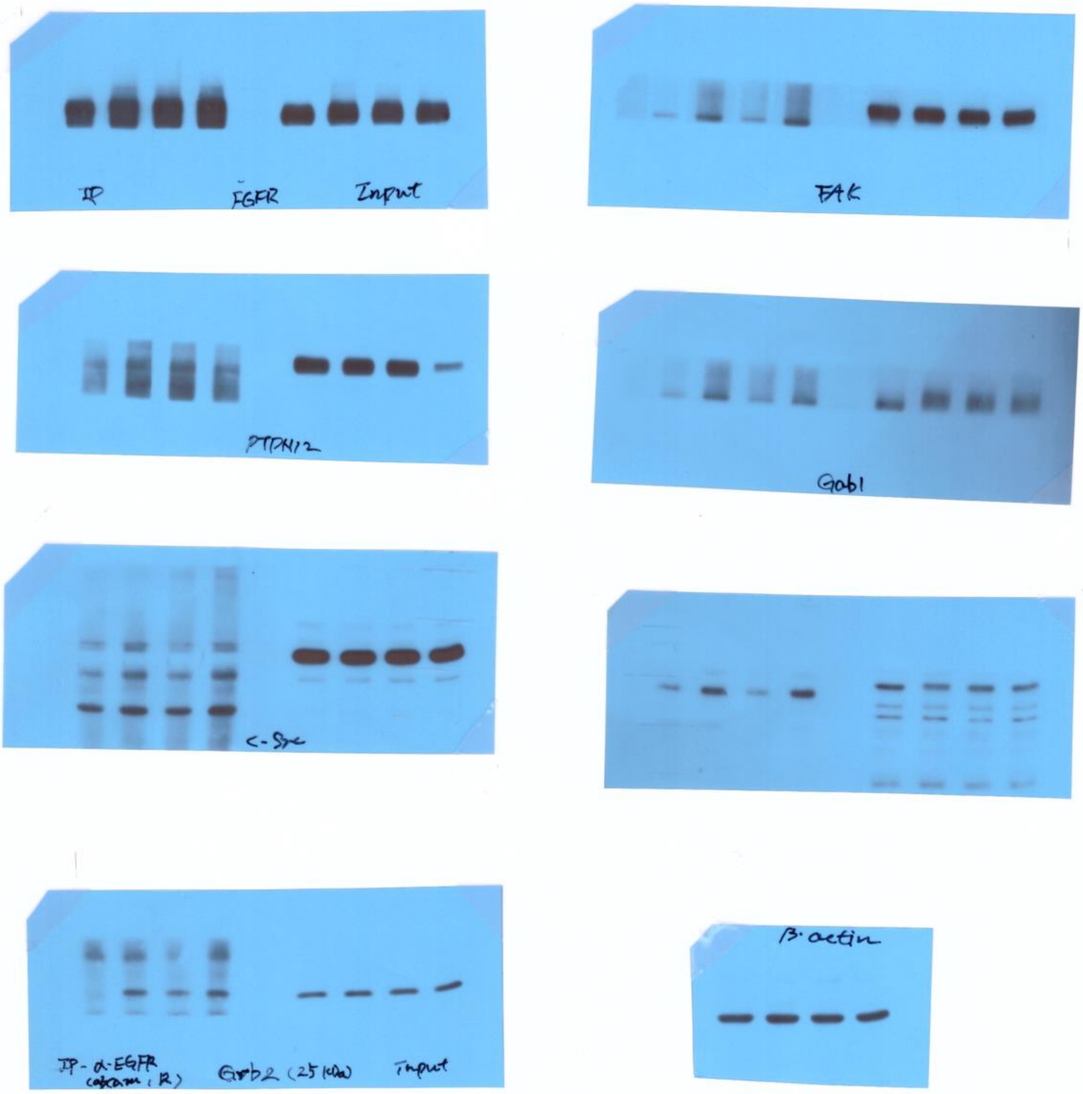
IP
EGFR
Input
FAK
PTPN12
Gab1
c-Src
IP-α-EGFR
(abcam, R)
Grb2 (25 kDa)
Input
β-actin

<Figure 6>

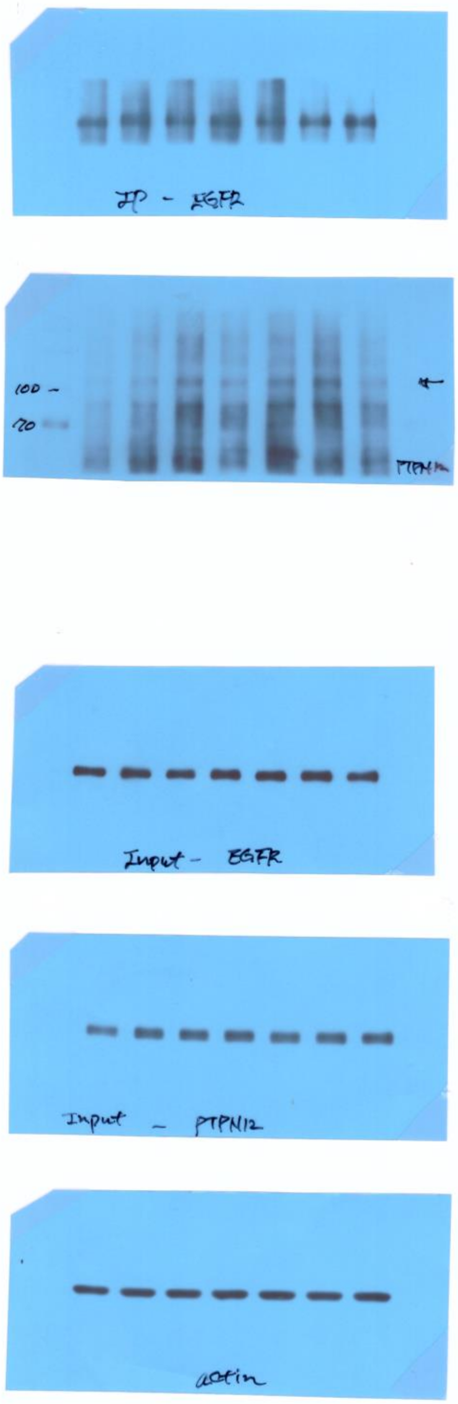

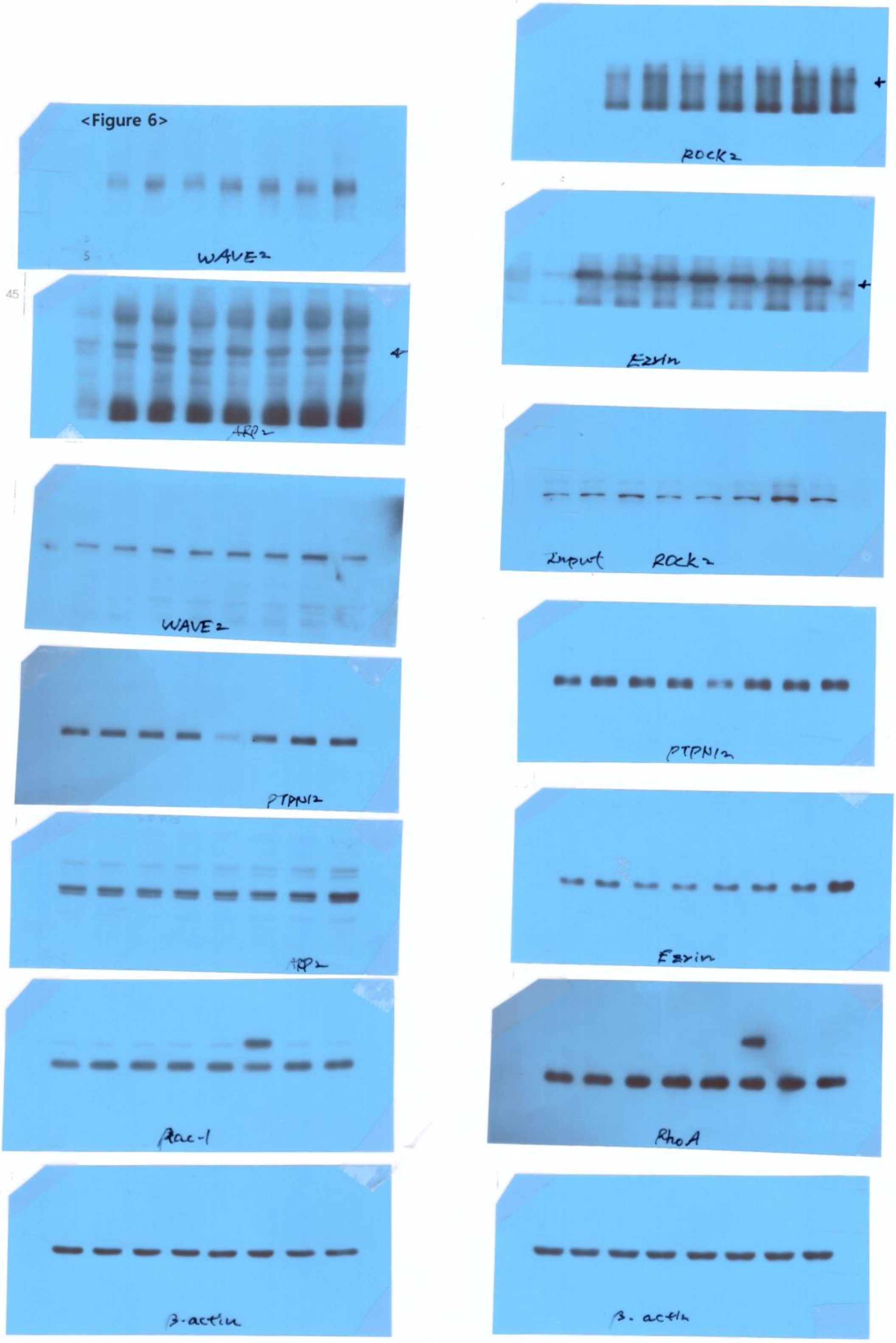
<Figure 6>
WAVE2
ARP2
WAVE2
PTPN12
ARP2
Rac-1
β-actin
ROCK2
Ezrin
Input ROCK2
PTPN12
Ezrin
RhoA
β-actin

<Figure 6>

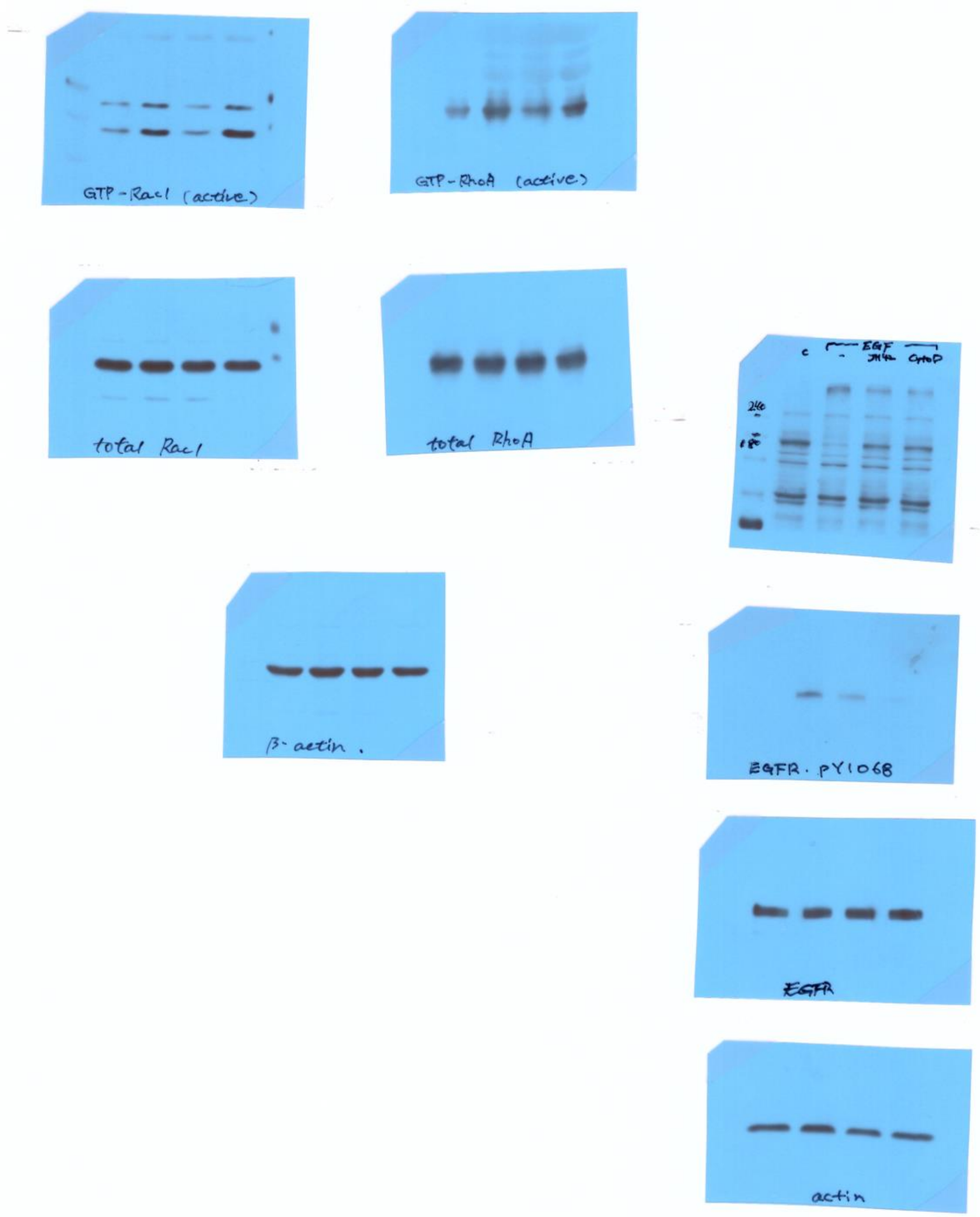
GTP-Rac1 (active)
GTP-RhoA (active)
total Rac1
total RhoA
β-actin
EGF
EGFR. pY1068
EGFR
actin

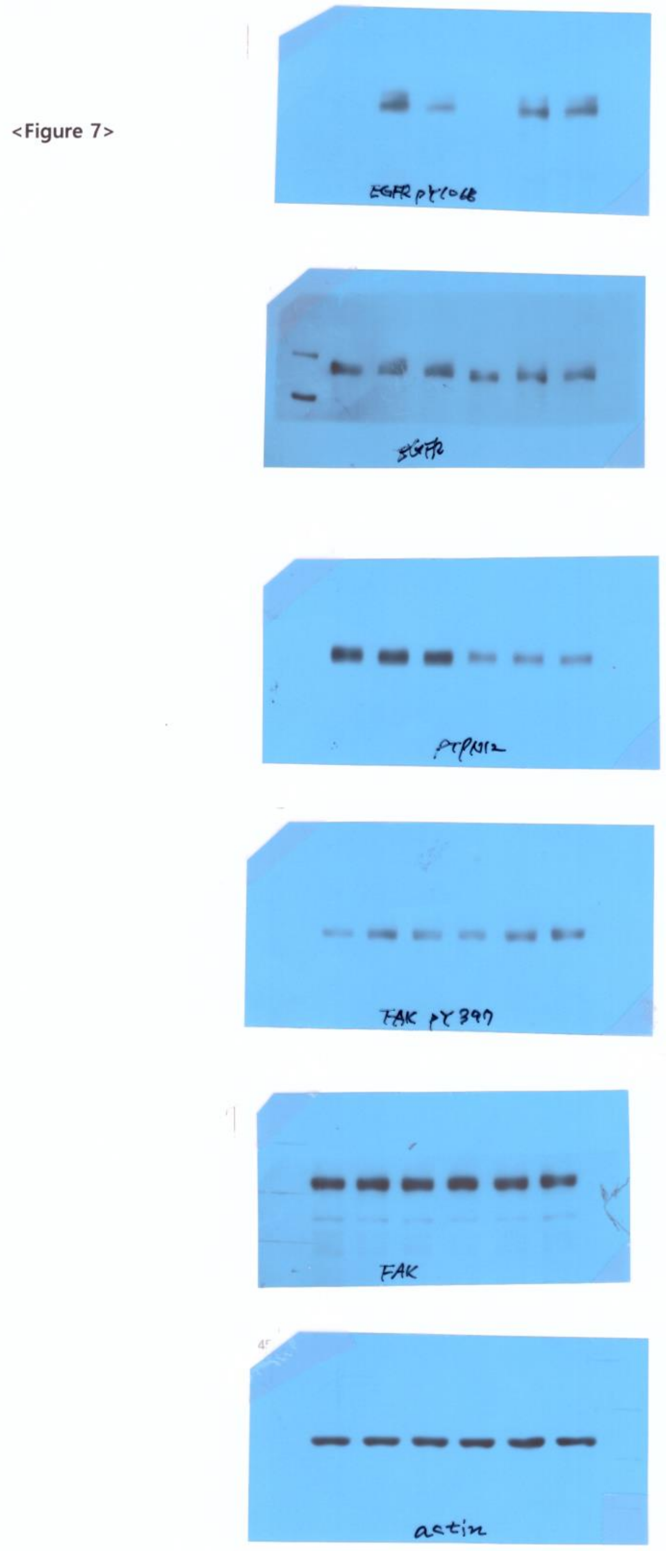

Figure S8. Uncropped western blot figures.

© 2020 by the authors. Licensee MDPI, Basel, Switzerland. This article is an open access article distributed under the terms and conditions of the Creative Commons Attribution (CC BY) license (http://creativecommons.org/licenses/by/4.0/).
